# Two Natural Flavonoid Substituted Polysaccharides from *Tamarix chinensis*: Structural Characterization and Anticomplement Activities

**DOI:** 10.3390/molecules27144532

**Published:** 2022-07-15

**Authors:** Yukun Jiao, Yiting Yang, Lishuang Zhou, Daofeng Chen, Yan Lu

**Affiliations:** School of Pharmacy, Institutes of Integrative Medicine, Fudan University, Shanghai 201203, China; 17111030034@fudan.edu.cn (Y.J.); 17211030024@fudan.edu.cn (Y.Y.); 20111030064@fudan.edu.cn (L.Z.)

**Keywords:** *Tamarix chinensis* Lour., flavonoid substituted polysaccharides, structural characterization, anticomplement activity, quercetin

## Abstract

Two novel natural flavonoid substituted polysaccharides (MBAP-1 and MBAP-2) were obtained from *Tamarix chinensis* Lour. and characterized by HPGPC, methylation, ultra-high-performance liquid chromatography-ion trap tandem mass spectrometry (UPLC-IT-MS^n^), and NMR analysis. The results showed that MBAP-1 was a homogenous heteropolysaccharide with a backbone of 4)-β-d-Glc*p*-(1→ and →3,4,6)-β-d-Glc*p*-(1→. MBAP-2 was also a homogenous polysaccharide which possessed a backbone of →3)-α-d-Glc*p*-(1→, →4)-β-d-Glc*p*-(1→ and →3,4)-β-d-Glc*p*-2-OMe-(1→. Both the two polysaccharides were substituted by quercetin and exhibited anticomplement activities in vitro. However, MBAP-1 (CH_50_: 0.075 ± 0.004 mg/mL) was more potent than MBAP-2 (CH_50_: 0.249 ± 0.006 mg/mL) and its reduced product, MBAP-1R (CH_50_: 0.207 ± 0.008 mg/mL), indicating that multiple monosaccharides and uronic acids might contribute to the anticomplement activity of the flavonoid substituted polysaccharides of *T. chinensis*. Furthermore, the antioxidant activity of MBAP-1 was also more potent than that of MBAP-2. In conclusion, these two flavonoid substituted polysaccharides from *T. chinensis* were found to be potential oxidant and complement inhibitors.

## 1. Introduction

The complement system, as the first defense line of human immune system, plays an irreplaceable role in numerous diseases [1]. Our previous study showed that the overactivation of complement system is involved with H1N1 induced pneumonia in mice [2]. Moreover, the significant elevation of peripheral complement has been recognized as a hallmark of respiratory distress syndrome associated with severe sepsis, cytokine storm, and multiple organ failure [3]. The autopsy results of COVID-19 patients also suggested that hyper complementation in plasma resulted in alveolar capillary wall damage with increased vascular permeability and further enhanced release of inflammatory mediators, and then intensified tissue damage [4]. In addition, two complement inhibitors, eculizumab and compstatin, have been suggested as potential treatments for COVID-19 [5]. In our previous research, anticomplement polysaccharides and flavonoids from several medicinal plants could significantly alleviate lung injury and increase the survival rate of H1N1 induced mice, and were non-toxic [2,6,7]. Hence, the medicinal plants provided a new resource of complement inhibitors for the treatment of viral pneumonia.

*Tamarix chinensis* Lour. (Tamaricaceae) has been widely used to treat rheumatoid arthritis, measles (Chinese Pharmacopoeia, 2020), and measles complicated with pneumonia [8]. Its flavonoids, triterpenoids, organic acids, and volatile oils showed anti-inflammatory, bacteriostatic, antioxidant, and hepatoprotective effects [9]. However, there were no reports about *T. chinensis* polysaccharides. In our preliminary studies, the crude polysaccharides of *T. chinensis*, MBAP90, showed significant anticomplement activity with CH_50_ value of 0.186 ± 0.003 mg/mL. Interestingly, MBAP90 exhibited the characteristic color reaction of flavonoids even after deproteinization and dialysis (cutting off M_W_: 5000 Da). The ^1^H-NMR signals at δ 6.5–8.0 also indicated that MBAP90 might contain flavonoid substituted polysaccharides.

In recent years, numerous methods have been applied to graft flavonoids and polysaccharides [10]. Some synthesized flavonoid-polysaccharide conjugates possessed unique advantages compared with flavonoids or polysaccharides. For example, quercetin-grafted carboxymethyl chitosan was amphiphilic with a low critical micelle concentration and good stability [11]. Quercetin-grafted hyaluronic was designed as tumor cell-targeted prodrug for its significant intestinal permeability, oral bioactivity and antitumor efficacy [12]. However, there were no reports of natural flavonoid substituted polysaccharides.

To explore anticomplement polysaccharides in *T. chininsis* and their anticomplement activities, MBAP90 was further purified by DEAE-cellulose and Sepharyl S-200, which led to the isolation of two novel homogenous polysaccharides, MBAP-1 and MBAP-2. This paper describes their structural characterization and anticomplement activities. As oxidative stress is vital for inflammatory responses in viral pneumonia, antioxidant activities were also determined herein as well [13].

## 2. Results

### 2.1. Isolation and Purification of MBAP-1 and MBAP-2

**Figure 1:**
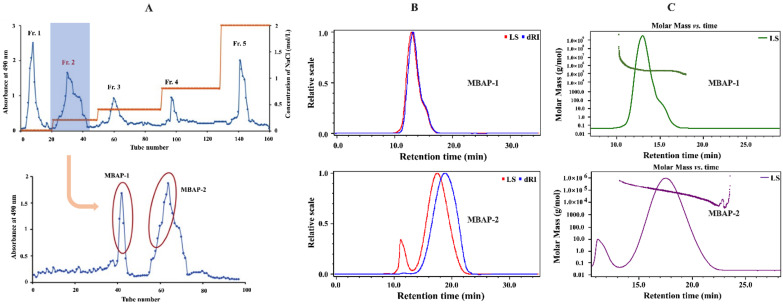
The eluted profiles and HPSEC-MALLS-RI results. (**A**) The eluted profile of MBAP90 on DEAE-52 column and Fr. 2 on Sepharyl S-200 column. (**B**) Superimposed spectra detected using RI and LC at angle of 90° on HPSEC-MALLS-RI. (**C**) Molar mass distribution detected by HPSEC-MALLS-RI.

MBAP-1 and MBAP-2 were isolated from the most potent anticomplement fraction (Fr. 2) of MBAP90, and the yields were 0.14% and 0.61%, respectively. The detailed elution curves are shown in Figure 1A.

### 2.2. Homogeneity and Molecular Weight Assessment

The homogeneity was evaluated by HPGPC-ELSD. As shown in Figure S1 (Supplementary Materials), MBAP-1 and MBAP-2 both showed one single narrow peak, indicating that they were both homogenous. As displayed in Figure 1B,C, the two polysaccharides were both further confirmed to be homogenous using HPSEC-MALLS-RI. Moreover, the results suggested that the relative molecular weights of MBAP-1 and MBAP-2 were 269.3 and 46.5 kDa, respectively. The detailed parameters of MBAP-1 and MBAP-2 were summarized in Table 1.

### 2.3. Monosaccharide Composition and Absolute Configuration Analysis

**Figure 2:**
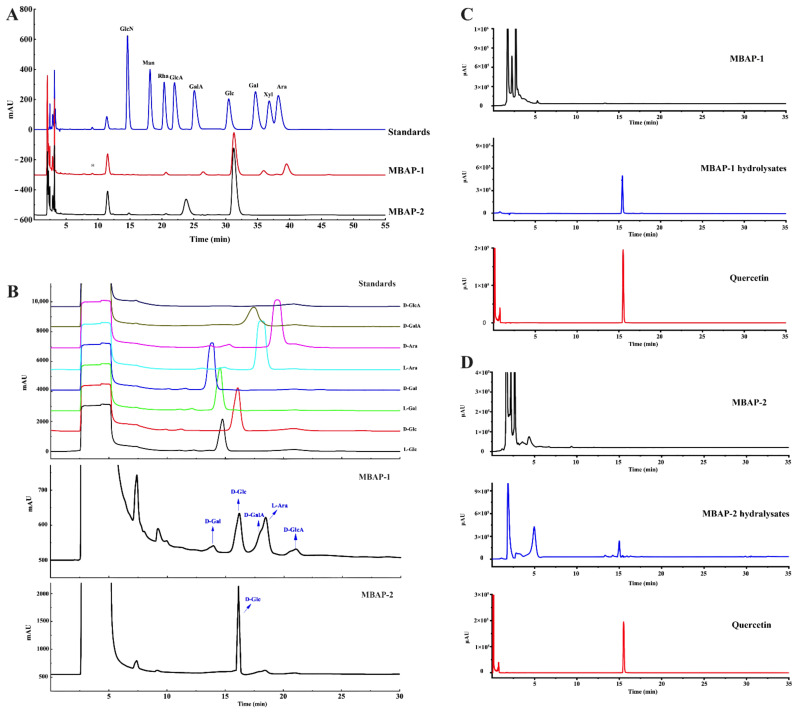
The chromatograms of monosaccharide composition (**A**), monosaccharide absolute configuration analysis (**B**) and UPLC-MS identification results of substituted flavonoids (**C**,**D**) of MBAP-1 and MBAP-2.

The monosaccharide composition results of MBAP-1 and MBAP-2 are presented in Figure 2A. Obviously, MBAP-1 was composed of glucose, galactose, arabinose, glucuronic acid, and galacturonic acid with the molar ratio of 54.54:4.21:18.18:4.87:4.21. MBAP-2 was mainly consisted of glucose. However, an unknown peak was presented at 24.67 min, which was further identified by GC-MS. The unknown monosaccharide was attributed as 2-*O*-methyl glucose by ion fragments of its alditol acetate (Figure S2). Thus, MBAP-2 was mainly composed of glucose and 2-*O*-methyl glucose with a molar ratio of 88.41:11.59. The *w*/*w* (%) ratio of each monosaccharide in two polysaccharides is presented in Table 2.

The monosaccharide absolute configurations were also analyzed. As shown in Figure 2B, the monosaccharides of MBAP-1 included d-glucose, d-galactose, l-arabinose, and d-galacturonic acid compared with the standard monosaccharides. Similarly, MBAP-2 consisted of d-glucose and 2-*O*-methyl-d-glucose.

### 2.4. FT-IR Spectroscopy Assessments

The FT-IR results of MBAP-1 and MBAP-2 are presented in Figure S3. The intense and broad peaks at 3267 and 3307 cm^−1^ indicated the stretching vibrations of hydroxyl groups of MBAP-1 and MBAP-2, respectively. The absorptions at 2924 and 2938 cm^−1^ were assigned to C-H stretching vibration, and the peak at 1593 cm^−1^ in MBAP-1 was due to the asymmetric C=O stretching vibration. The peaks at 1378 and 1405 cm^−1^ and shoulder absorptions were assigned to C-H bending vibration [14]. The typical absorptions of the pyranose ring of polysaccharide presented at 615 or 765 and 632 cm^−1^ [15]. The sharp peaks at 1031, 1078 and 1106 cm^−1^ were stretching vibrations of C-O-C [14].

### 2.5. Methylation Analysis

Methylation could provide the fundamental glycosidic linkages [16]. The detailed methylation results of MBAP-1, MBAP-1R and MBAP-2 are summarized in Table 3. The uronic acidic residues were confirmed by the comparison of MBAP-1 and MBAP-1R. MBAP-1 was composed of seven kinds of partially methylated alditol acetates (PMAAs): 2-*O*-methyl-d-glucitol, 4,6-di-*O*-methyl-d-glucitol, 2,3-di-*O*-methyl-l-arabinitol, 2,3,5-tri-*O*-methyl-l-arabinitol, 2,6-di-*O*-methyl-d-glucitol, 2,3,6-tri-*O*-methyl-d-galactitol and 2,3,6-tri-*O*-methyl-d-glucitol. While two new PMAAs of 2,3,4,6-tetra-*O*-methyl-d-glucitol and 2,3,4,6-tetra-*O*-methyl-d-galactitol were presented in MBAP-1R, indicating they might be uronic acidic residues. MBAP-2 was composed of 2,4-di-*O*-methyl-d-glucitol, 2,3,4-tri-*O*-methyl-d-glucitol, 2,6-di-*O*-methyl-d-glucitol, 2,3,6-tri-*O*-methyl-d-glucitol, and 2,3,4,6-tetra-*O*-methyl-d-glucitol. More detailed information concerning the residues was confirmed by NMR analysis.

### 2.6. Identification of Substituted Flavonoid with UPLC-IT-MS^n^ and NMR Analysis

The unpredicted signals between δ 6.5–7.5 in ^1^H-NMR spectra (Figures S6A and S7A) indicated that non-polysaccharide constituents might be existed in MBAP-1 and MBAP-2. Under consideration of their perfect homogeneity, it was speculated that the non-polysaccharide components were linked with the polysaccharide chains. To identify the non-polysaccharide part, UPLC-IT-MS^n^ was applied for further identification. As shown in Figure 2C, compared with MBAP-1, an obvious peak was presented in its full hydrolysates. The ions of *m*/*z* 301.05 [M − H]^−^ and *m*/*z* 303.07 [M + H]^+^ suggested its relative molecular weight might be 302.04 Da. And typical fragmental ions of *m*/*z* 179.00 and 151.00 in negative mode indicated it might be quercetin [17]. Compared with the chromatogram and fragmental ions of quercetin CRS, quercetin was confirmed in MBAP-1. The flavonoid in MBAP-2 was similarly identified to be quercetin as well (Figure 2D). Meanwhile, the contents of quercetin in MBAP-1 and MBAP-2 were 12.03% and 15.96%.

**Figure 3:**
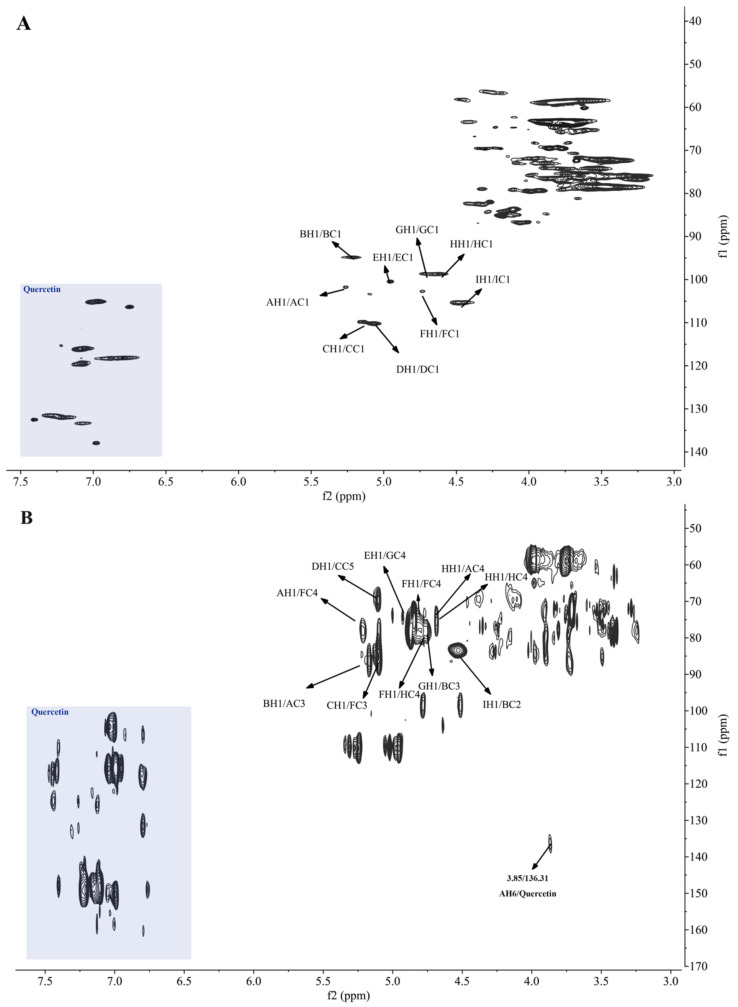
HSQC (**A**) spectrum and HMBC (**B**) spectrum of MBAP-1.

**Figure 4:**
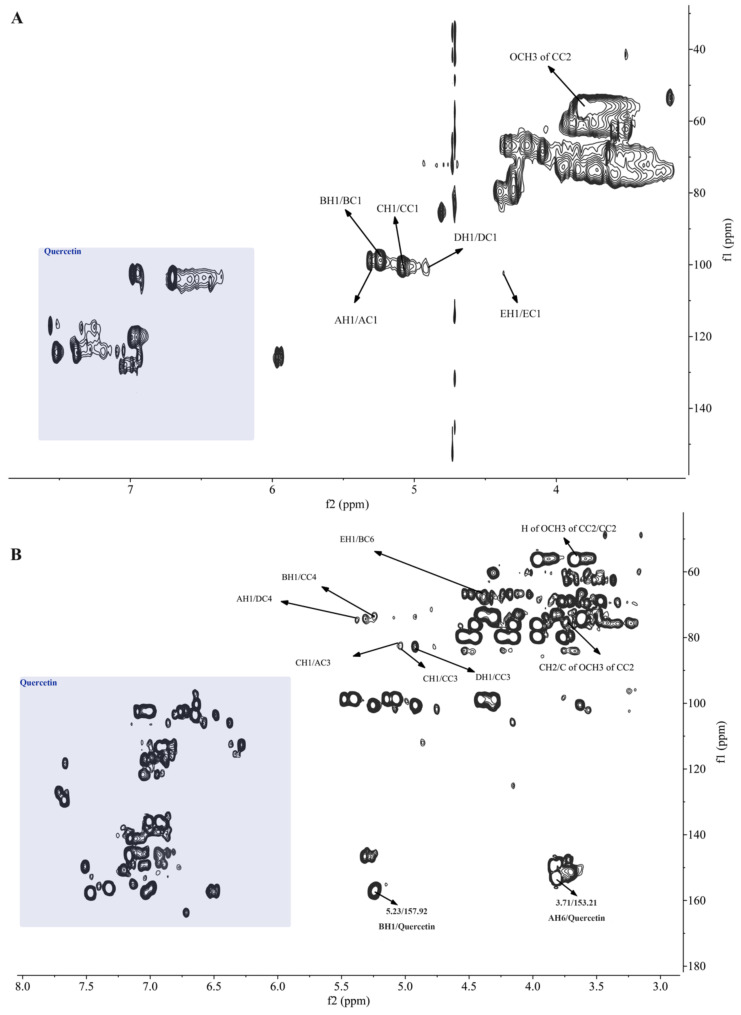
HSQC (**A**) spectrum and HMBC (**B**) spectrum of MBAP-2.

Furthermore, the existence of quercetin in MBAP-1 and MBAP-2 was confirmed by the NMR data. As we all know, the signals of quercetin commonly presented at δ 6.0–8.0 in ^1^H-NMR spectrum and δ 90–180 in ^13^C-NMR spectrum [18], most of which were in lower field regions and could be easily distinguished from polysaccharides’ signals. The ^13^C-NMR signals at 177.32 in MBAP-1 (Figure S6B) and 175.57 in MBAP-2 (Figure S7B) were conclusively assigned to carbonyl carbon at C-4 of quercetin. As shown in the grey area of Figure 3 and Figure 4, the signals in lower field mainly belonged to quercetin in the polysaccharides. However, compared with the reported data [18], signals of quercetin herein were slightly shifted, which might be induced by different NMR solvents and spatial effects from polysaccharides. The effect of different solvents on chemical shifts of quercetin was further confirmed by Figure S8. As expected, most of the NMR shifts of quercetin in the mixture of d_6_-DMSO and D_2_O (1:1) were higher than the corresponding signals in pure d_6_-DMSO.

### 2.7. NMR Analysis of Glycosidic Residues

NMR is very useful in the characterization of anomeric configurations (α- or β-), patterns and sequences of glycosidic linkages. As shown in Figure 3A and Figure S6B, the signals at δ 5.13/109.84 and 5.07/110.23 in HSQC spectrum of MBAP-1 were obviously observed, indicating they might be the anomeric signals of arabinose residues because the anomeric carbon signals of arabinose residues were generally at δ 105–110. GC-MS results indicated MBAP-1 consisted of →5)-α-l-Ara*f*-(1→ and α-l-Ara*f*-(1→, and the anomeric carbon signal of α-l-Ara*f*-(1→ was commonly in lower field than that of →5)-α-l-Ara*f*-(1→ [19]. Thus, the signals at δ 5.13/109.84 and 5.07/110.23 (Figure 3A) were assigned to →5)-α-l-Ara*f*-(1→ and α-l-Ara*f*-(1→, and named as residues C and D, respectively. In addition, the cross signals at δ 5.07/84.16 (DH1/DC2), 4.12/110.23 (DH2/DC1), 4.12/76.51 (DH2/DC3) and 3.88/84.16 (DH3/DC2) in HMBC spectrum and δ 4.12/84.16 (DH2/DC2) and 3.88/76.51 (DH3/DC3) in HSQC spectrum suggested the chemical shifts of H2/C2 and H2/C3 of α-l-Ara*f*-(1→ was δ 4.12/84.16 and 3.88/76.51, respectively. With the same approach, the chemical shifts of H4/C4 and H5/C5 were assigned as δ 4.06/86.96 and 3.72/63.41, respectively. Consequently, the whole chemical shifts of α-l-Ara*f*-(1→ were assigned and further compared with reported literatures [20,21]. For the overlap of anomeric carbon peaks at 90–105 ppm in ^13^C-NMR spectrum, the anomeric signals of other residues were analyzed with HSQC spectrum (Figure 3A). The intense signal at δ 4.62/98.70 was attributed to →4)-β-d-Glc*p*-(1→ for its abundance in MBAP-1 and noted as residue H. According to the ^1^H-NMR, ^13^C-NMR, HSQC, HMBC spectra and reported data [22], δ 4.62 (H1) was correlated to δ 98.70 (HC1) in HSQC and 72.40 (HC2) and 74.20 (HC3) in HMBC spectrum, and δ 3.92 (HH4) was correlated to δ 74.20 (HC3), 71.68 (HC5), and 58.92 (HC6) in HMBC spectrum. Thus, the whole chemical shifts of →4)-β-d-Glc*p*-(1→ were assigned and compared with published data [22]. Accordingly, based on the methylation results and NMR data, the anomeric signals at δ 5.26/101.76, 5.21/94.86, 4.95/100.47, 4.73/102.72, 4.70/98.66, and 4.48/105.32 in HSQC spectrum were assigned to →3,4,6)-α-d-Glc*p*-(1→, →2,3)-α-d-Glc*p*-(1→, β-d-Gal*p*A-(1→, →3,4)-β-d-Glc*p*-(1→, →4)-β-d-Gal*p*-(1→ and β-d-Glc*p*A-(1→, and denoted as A, B, E, F, G, and I, respectively. Then, the complete chemical shifts of glycosidic residues of MBAP-1 were confirmed by HSQC and HMBC spectra, as summarized in Table 4, and were also consistent with reported literature [23,24,25,26,27,28].

The monosaccharide composition result showed that MBAP-2 was a polysaccharide mainly composed of glucose. Based on the literature [27,28,29,30,31] and the results of GC-MS, the signals at δ 5.32/98.91, 5.23/98.68, 5.08/100.60, 4.92/100.26 and 4.37/102.53 (Figure 4A) (labeled as residue A, B, C, D, and E) were attributed to →3,6)-α-d-Glc*p*-(1→, →6)-α-d-Glc*p*-(1→, →3,4)-α-d-Glc*p*-(1→, →4)-β-d-Glc*p*-(1→ and β-d-Glc*p*-(1→, respectively. The chemical shifts of glycosidic residues of MBAP-2 were confirmed by HSQC and HMBC spectra and similarly assigned with the method above (Table 4). As shown in Figure 4 and Figure S7, the signal at δ 3.71/55.20 in HSQC spectrum and δ 3.71/75.51 in HMBC spectrum demonstrated the presence of OCH_3_ [32], which was linked to C-2 of residue C in MBAP-2.

### 2.8. NMR Analysis of Linkages and Sequences

**Figure 5:**
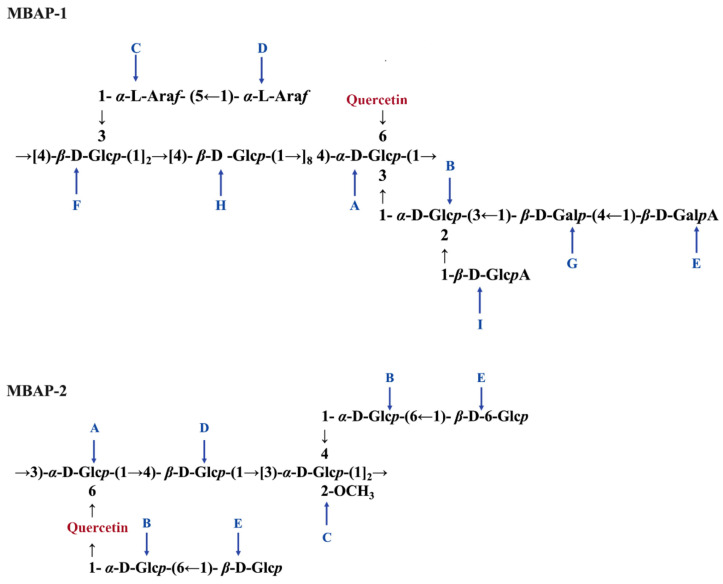
The putative structures of the repeating units of MBAP-1 and MBAP-2. The residues were labeled as A, B, C, D, E, F, G, H and I.

The sequences and linkage sites among glycosidic residues were confirmed by HMBC analysis. As displayed in Figure 3B, the fiercely cross signal at δ 4.62/78.55 (HH1/HC4) indicated O-1 of residue H was linked to C-4 of residue H in MBAP-1. And the GC-MS results suggested that the residue H accounted for 41%, indicating it was an important part of backbone chain. In addition, the strong signals at δ 4.73/78.55 (FH1/HC4) and 4.62/77.49 (HH1/AC4) revealed that O-1 of residue F was linked to C-4 of residue H and O-1 of residue H was linked to C-4 of residue A. Moreover, the cross peak at δ 5.26/76.40 (AH1/FC4) suggested that O-1 of residue A was linked to C-4 of residue F, and it also indicated that the backbone chains of MBAP-1 was residue A, repeating residue H and residue F in sequence. Accordingly, the signals at δ 4.73/76.40 (FH1/FC4), 5.13/86.66 (CH1/FC3), 5.07/68.53 (DH1/CC5), 5.21/86.21 (BH1/AC3), 4.48/84.89 (IH1/BC2), 4.70/80.62 (GH1/BC3) and 4.95/76.18 (EH1/GC4) suggested that O-1 of residue F was linked to C-4 of residue F; O-1 of residue C was linked to C-3 of residue F; O-1 of residue D was linked to C-5 of residue C; O-1 of residue B was linked to C-3 of residue A; O-1 of residue I was linked to C-2 of residue B; O-1 of residue G was linked to C-3 of residue B; O-1 of residue E was linked to C-4 of residue G. As shown in Figure 3B, the cross peak at δ 3.85/136.31 was obviously observed, indicating that O-6 of residue A was linked to quercetin. Consequently, the proposed primary structure of MBAP-1 is shown in Figure 5.

Similarly, the cross peaks at δ 5.32/74.82 (AH1/DC4), 5.23/74.51 (BH1/CC4), 5.08/81.47 (CH1/AC3), 4.92/74.51 (DH1/CC3), and 4.37/68.56 (FH1/BC6) in the HBMC spectrum of MBAP-2 (Figure 4B) indicated the linkage between O-1 of residue A and C-4 of residue D, between O-1 of residue B and C-4 of residue C, between O-1 of residue C and C-3 of residue A, between O-1 of residue D and C-3 of residue C, between O-1 of residue E and C-6 of residue B. The cross signal at 5.08/82.47 (CH1/CC3) and molar ratio result in GC-MS indicated O-1 of residue C was linked at C-3 of residue C and repeated three times. As previous literature reported, the chemical shifts of C-6 and C-8 were lower than C-2′, C-5′ and C-6′ in quercetin [18]. Several cross signals were observed at quercetin signal area in the HSQC spectrum (Figure 4A) and the signals at δ 6.36/102.50 and 6.70/105.36 might belonged to ring A of quercetin for their relevant lower chemical shifts. Moreover, the correlation signals at δ 6.36/157.92 and 6.86/153.21 in the HMBC spectrum (Figure 4B) indicating the signals at δ 157.92 and 153.21 might belong to ring A of quercetin. Thus, the cross signals at δ 5.23/157.92 and 3.71/153.21 in HMBC spectrum indicating that O-1 of residue B and O-6 of residue A were linked to quercetin. Above all, the possible repeating units of MBAP-2 were displayed in Figure 5.

### 2.9. Morphological Analysis

**Figure 6:**
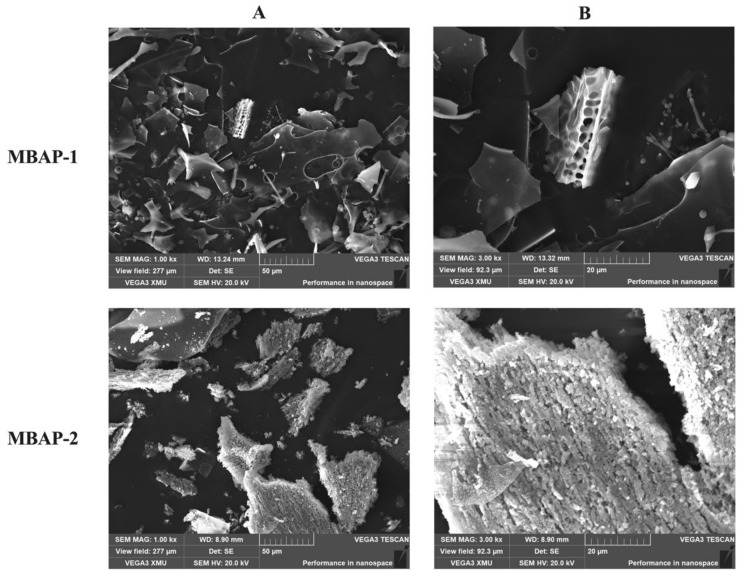
SEM results of MBAP-1 and MBAP-2 at different magnifications ((**A**): 1000×, (**B**): 3000×).

SEM has become more commonly applied to directly observe the microstructures and aggregation properties of polysaccharides. The morphological pictures of MBAP-1 and MBAP-2 were obtained and shown in Figure 6. MBAP-1 macroaggregated state presented as irregularly curled sheets with smooth surface and spatially as holes of varying sizes. The stacks between sheets were irregular and varied in size, which was consistent with the primary structure of MBAP-1. MBAP-2 appeared as sheets of varying size, with a flocculent surface, and more regularly arranged and loose accumulations at the magnification of 3000×, which was consistent with fewer branches and shorter chains in its primary structure.

### 2.10. AFM Analysis

AFM is a powerful technique to observe the direct macromolecular morphology of polysaccharides dissolved in water without interacted effects, which could help us to better understand the natural parameters of polysaccharides [33]. In certain conditions, scanning tunneling microscope could even characterize the structures of polysaccharides [34]. As shown in Figure S9, they were aggregated loosely as a cloudy mass, with pores in the middle. Compared with MBAP-2, MBAP-1 showed looser and more porous, which was consistent with its larger molecular weight and more branches, which was also consistent with their SEM images and primary structures.

### 2.11. Anticomplement Activity

**Figure 7:**
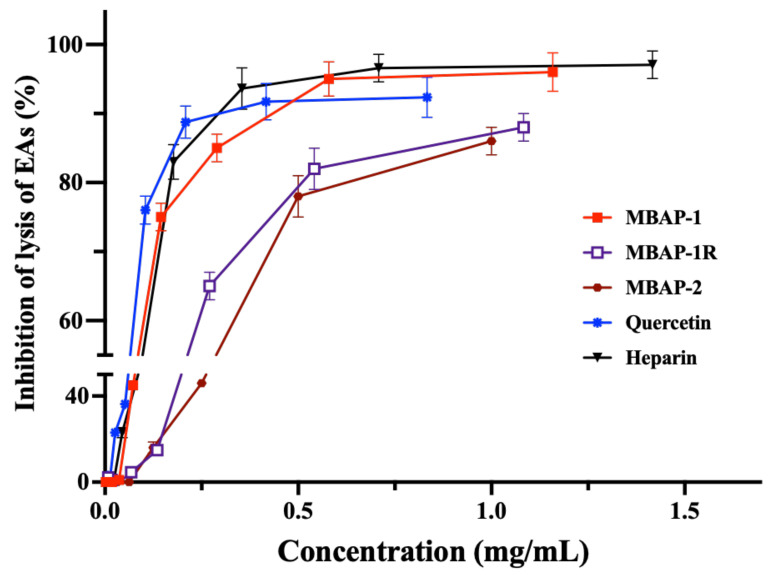
Anticomplement activities of MBAP-1, MBAP-2, MBAP-1R and quercetin.

The anticomplement activities of MBAP-1 and MBAP-2, as well as MBAP-1R and quercetin were evaluated in vitro by hemolytic assay [15]. As shown in Figure 7, MBAP-1 and quercetin presented excellent hemolytic inhibition through classic pathway with CH_50_ values of 0.075 ± 0.004 and 0.085 ± 0.005 mg/mL, respectively, which were comparable to that of heparin (CH_50_: 0.079 ± 0.003 mg/mL). MBAP-2 (CH_50_: 0.249 ± 0.006 mg/mL) and MBAP-1R (CH_50_: 0.207 ± 0.008 mg/mL) showed moderate anticomplement activities. This indicated that different polysaccharide components might result in their different anticomplement activities, even though they were both quercetin substituted. Moreover, the activity of MBAP-1 was weakened when its uronic acids were reduced.

### 2.12. Antioxidant Activity

**Figure 8:**
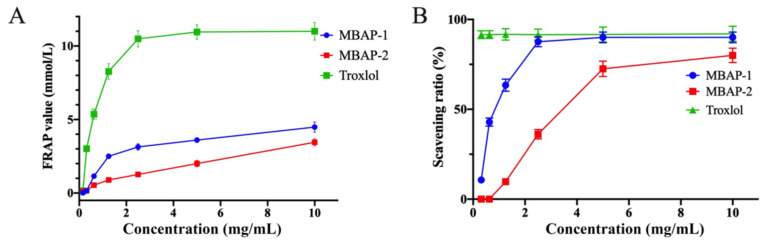
Antioxidant activities of MBAP-1 and MBAP-2. (**A**) The FRAP results of MBAP-1 and MBAP-2. (**B**) The ABTS results of MBAP-1 and MBAP-2.

The antioxidant activities of MBAP-1 and MBAP-2 were evaluated in vitro by the ABTS and FARP methods [35]. As displayed in Figure 8A, FRAP values of MBAP-1 were from 0.0145 ± 0.002 mmol/L to 4.483 ± 0.348 mmol/L, while that of MBAP-2 were from 0.124 ± 0.012 mmol/L to 3.455 ± 0.230 mmol/L. As shown in Figure 8B, both MBAP-1 and MBAP-2 exhibited dose-dependent antioxidant activity in the concentration range of 0.31–10.00 mg/mL. The IC_50_ values of MBAP-1 and MBAP-2 were calculated to be 0.935 ± 0.050 mg/mL and 3.286 ± 0.030 mg/mL, respectively. The above results suggested that both MBAP-1 and MBAP-2 exhibited antioxidant activity, and MBAP-1 with multiple monosaccharide composition showed slightly stronger activity.

## 3. Discussion

Polysaccharides are macromolecules and present various beneficial bioactivities which are directly influenced by their relative molecular weights, uronic acids, and monosaccharide composition [36]. Our previous works showed that polysaccharides with richer monosaccharide composition, higher uronic acid content, or more branches could exhibit stronger anticomplement activity [16,37]. As expected, MBAP-1, with lager molecular weight (263.9 kDa) and multiple monosaccharides, exhibited much more potent anticomplement activity than MBAP-2, which was with smaller molecular weight and had one monosaccharide. Compared with MBAP-1, its reduced product (MBAP-1R) showed obviously weaker activity, revealing that uronic acids played an indispensable role in anticomplement activity. In general, glucan has no anticomplement activity [38]. The moderate activity of MBAP-2 should be owing to its quercetin component, which exhibited potent anticomplement activity. Similarly, it had been demonstrated that the strong antioxidant activity of polysaccharides was related to the composition of galactose and uronic acid β-glycosidic linkages [39], which was consistent with the stronger antioxidant activity of MBAP-1.

In recent years, flavonoid-polysaccharide conjugates have attracted more and more attention for their improved activities or expanded properties in drug delivery attributed to the synergistic effects of the two different components. It was reported that catechin-grafted chitosan showed improved adsorption, controlled release abilities on dye, and increased antioxidant activity compared to chitosan [40]. Quercetin-grafted hyaluronic exhibited significant and sustained pH dependent drug release behaviors and higher selectivity to CD4 cell than free quercetin solution [12]. Moreover, flavonoid grafted onto pectin significantly improved the antioxidant activity of natural pectin [41]. MBAP-1 and MBAP-2 were both quercetin substituted polysaccharides and possessed potent anticomplement and antioxidant activities. They are supposed to have beneficial effects on viral pneumonia based on the potential synergistic mechanism of the active flavonoid and polysaccharide components. Their in vivo effects and mechanisms on H1N1-induced acute lung injury in mice will be investigated and reported in the near future.

## 4. Materials and Methods

### 4.1. Materials

The leaves and twigs of *Tamarix chinensis* Lour. were purchased from Bozhou Chinese herb market (Anhui, China) and authenticated by Professor Yan Lu of Fudan University, Shanghai, China. A voucher specimen (TCL20180506) has been deposited at Department of Natural Medicine, School of Pharmacy, Fudan University, Shanghai, China. Information concerning chemical regents and bio-materials used for structural characterization, anticomplement and antioxidant activities is shown in Supplementary Method S1.

### 4.2. Activity-Guided Extraction, Isolation and Purification

The leaves and twigs of *T. chinensis* (10 kg) were defatted with 10 vols of 95% ethanol (100 L) three times (2 h each time) and extracted with 10 vols of water (100 L) three times (2 h each time). The water extracts were concentrated to 15 L and followed by graded ethanol precipitation at final concentrations of 80% and 90%. The extracts were further mixed with 20% trichloroacetic acid (*v*/*v*, 1:1) to deproteinize. After dialyzing with water for 72 h, crude polysaccharides, MBAP80 (239 g) and MBAP90 (120 g) were obtained. Because of the stronger anticomplement activity (CH_50_ of MBAP80: 0.889 ± 0.007 mg/mL, CH_50_ of MBAP90: 0.296 ± 0.006 mg/mL), MBAP90 (100 g) was further purified by chromatography on DEAE-cellulose, eluted with water and successive concentrations of NaCl (0.2, 0.4, 0.8 and 2.0 M). Moreover, the fraction eluted with 0.2 M NaCl (Fr. 2) showed the most potent anticomplement activity (CH_50_: 0.102 ± 0.006 mg/mL). It was further purified by Sepharyl S-200 to obtain two homogeneous polysaccharides, MBAP-1 (205 mg) and MBAP-2 (920 mg), respectively. The contents of total carbohydrate, uronic acid and protein of MBAP-1 and MBAP-2 were measured according to reported methods [6] and replicated three times.

### 4.3. Homogeneity and Molecular Weight Determination

The homogeneity and molecular weights of MBAP-1 and MBAP-2 were analyzed by HPGPC-ELSD and HPSEC-MALLS-RI. More details are shown in Supplementary Method S2.

### 4.4. Monosaccharide Composition and Absolute Configuration Analysis

The monosaccharide composition of MBAP-1 and MBAP-2 was determined using HPLC on an Agilent 1260 HPLC system (Agilent Technologies, Santa Clara, CA, USA) by the method reported before [42]. Briefly, the samples were fully hydrolyzed with TFA (2.5 M) and derivatized with PMP. The PMP-derivates were separated on an Agilent Eclipse Plus C18 column (Agilent Technologies, Santa Clara, CA, USA) (4.6 × 250 mm, 5 μm) at a flow rate of 1.0 mL/min with the mobile phase of a mixture of phosphate buffered saline (0.1 M, pH 6.7) and acetonitrile (83:17), and detected under the wavelength of 245 nm.

To identify the unknown monosaccharide in MBAP-2, MBAP-2 was hydrolyzed with TFA, then converted into alditol acetates according to the methods described in Section 4.5 and then analyzed by GC-MS with a HP-5MS column (0.25 mm × 30 m, 0.25 µm). More details are shown in Supplementary Method S3.

The monosaccharide configuration of MBAP-1 and MBAP-2 was determined according to the literature [43]. Briefly, the sample was hydrolyzed, and reacted with l-cysteine methyl ester and pyridine, follow by *O*-tolyisothiocyanate. The reaction products were analyzed on an Agilent 1260 HPLC system equipped with the Agilent Eclipse Plus C18 column (4.6 × 250 mm, 5 μm). It was eluted with the mixture of 0.1% acetic acid in water-acetonitrile (25:75) at a flow rate of 0.8 mL/min and detected under the wavelength of 250 nm.

### 4.5. Reduction, Methylation and GC-MS Analysis

The methylation of MBAP-1 and MBAP-2 was performed with modified Hakomori method according to the literature [18]. Furthermore, in order to confirm the residues of uronic acid in MBAP-1, MABP-1 and its reduced product (MBAP-1R) were both determined. Detailed information is described in Supplementary Method S4.

### 4.6. Qualitative and Quantitative Analysis of Flavonoid Component

In this part, ultra-high-performance liquid chromatography-ion trap tandem mass spectrometry (UPLC-IT-MS^n^) was used to recognize the possible flavonoid component in the two polysaccharides. MBAP-1, MBAP-2 and their full hydrolysates were determined on a Dionex Ultimate 3000 UPLC system (Thermo Fisher Scientific, Waltham, MA, USA) equipped with a LTQ Velos Pro mass spectrometer (Thermo Fisher Scientific, Waltham, MA, USA) using a YMC-Triart C18 column (YMC Europe GmbH, Dinslaken, Germany) (150 mm × 2.1 mm, 1.9 μm). The column temperature and detection wavelength were set at 25 °C and 254 nm, respectively. The mobile phase consisted of water containing 0.1% formic acid (A) and acetonitrile containing 0.1% formic acid (B). An optimized elution gradient was set at follows: 0–2 min, 5% B; 2–35 min, 5–100% B. The injection volume and flow rate were 10 µL and 0.3 mL/min, respectively.

MS data was acquired in both positive and negative ion modes, and the device parameters were set as: auxiliary gas (N_2_) flow, 10 arb; sheath gas (N_2_) flow, 40 arb; collision gas (He); source voltage, 3.5 kV; capillary temperature, 350 °C; capillary voltage, 35 V. A data-dependent scan mode was selected for MS^n^ analysis. The flavonoid components were recognized by the positive and negative MS fragments and further confirmed by comparing with the reference substance.

The quantitative analysis was performed with colorimetry method [44] using quercetin as reference and replicated three times. Briefly, a series of concentrations (0, 0.016, 0.032, 0.048, 0.064 and 0.080 mg/mL) of quercetin and the full hydrolysates were reacted with AlCl_3_ (0.1%, 2 mL) in ethanol solution for 10 min, and then detected at 254 nm by a Multiskan Go microplate reader (Themo Fisher Scientific, Waltham, MA, USA). The content of quercetin was calculated based on the standard curve.

### 4.7. NMR and FT-IR Spectroscopy Analysis

The samples of MBAP-1 and MBAP-2 detected for NMR and FT-IR were prepared according to the method reported before [16].

Briefly, MBAP-1 and MBAP-2 were mixed with KBr, pressed into pellets, and then measured with the FT-IR spectrometer (PerkinElmer, Waltham, MA, USA) from 4000 to 400 cm^−1^ at a resolution of 2 cm^−1^ to obtain their FT-IR spectra.

For the NMR experiments, MBAP-1 and MBAP-2 (each 50 mg) were dissolved in D_2_O. Then, the ^1^H-NMR, ^13^C-NMR, HSQC and HMBC spectra were recorded at 24 °C with an NMR spectrometer (600 M, AVANCE III HD, Bruker, Brno, Switzerland). The detailed setting parameters are shown in the Supplementary Table S1.

### 4.8. Microstructure and Atomic Force Microscopy (AFM) Analysis

The micromorphology of MBAP-1 and MBAP-2 were examined with an environmental scanning electron microscope (SEM) (VEGA3 XMU, Tescan, Hitacchi, Czech Republic). Briefly, the dried powder was fixed on a silicon wafer, sprayed with gold powder via a sputter-coater under 10 kV acceleration voltage, and SEM images at different magnifications were obtained.

Furthermore, MBAP-1 and MBAP-2 were dissolved in deionized water and diluted to 1.0 ng/mL. Then, 5 µL of the solution was deposited onto a freshly cleaved mica surface and dried under infrared light. The samples were determined by Environment Control Scanning Probe Microscope (Nanonavi E-Sweep, Hitachi High-Technologies, Hitachi, Marunouchi, Japan) on tapping-mode.

### 4.9. Anticomplement Activity Evaluation

The anticomplement activity through the classical pathway (CP) was determined by hemolytic assay with our laboratory’s protocol [16]. Guinea pig sera (GPS) were used as complement source and heparin was used as positive control in this experiment. Hence, 2% SRBCs (sensitized sheep red blood cells) were prepared by washing with BBS three times and then diluted with BBS. The GPS (1:80) was chosen to give sub-maximal lysis in the absence of complement inhibitors. Various dilutions of the tested samples (200 µL) were mixed with 200 µL of GPS, 100 µL of EAs, and 100 µL anti-sheep erythrocyte antibody (1:1000 diluted). Then, the mixture was incubated at 37 °C for 30 min. After incubation, the mixtures were centrifuged immediately (3500 rpm, 4 °C) for 10 min. The optical density of the supernatant (200 µL) was measured at 405 nm by a Multiskan Go microplate reader (Themo Fisher Scientific Inc., Waltham, MA, USA). Inhibition rate of lysis induced by the test samples were calculated by the formula: Hemolytic inhibition rate (%) = [1 − (A_test_ − A_sample control_)/(A_100%lysis_ − A_vehicle control_] × 100%. The CH_50_ value was the sample concentration resulting in 50% hemolytic inhibition.

### 4.10. Antioxidant Activity Evaluation

The antioxidant activities were evaluated by ABTS and FARP analysis with Beyotime Elisa kits (Shanghai, China). The experiments were performed based on Elisa kit instructions.

## 5. Conclusions

Two novel natural flavonoid substituted polysaccharides were isolated and purified from *Tamarix chinensis* Lour. and they both exhibited potent anticomplement and antioxidant activities. Their primary structures were elucidated by monosaccharide composition, IR, d/l configuration, methylation, NMR, and UPLC-MS/MS analysis. In addition, the structure–activity relationship analysis demonstrated that uronic acids, monosaccharide composition, and quercetin were important for their anticomplement activities. In consideration of the advantages of flavonoid substituted polysaccharides in drug delivery and bioactivity, the quercetin substituted polysaccharides of *T. chinensis* might be better complement and oxidation inhibitors.

## Figures and Tables

**Table 1:** The molecular parameters of MBAP-1 and MBAP-2 determined by SEC-MALLS-RI.

Molecular Characteristics	Parameter	Detection Results	
		MBAP-1	MBAP-2
Polydispersity	Mw/Mn	1.01	1.07
	Mz/Mn	1.04	1.05
Molar mass moments (g/mol)	Mw	2.693 × 10^5^	4.650 × 10^4^
	Mn	2.537 × 10^5^	4.611 × 10^4^
	Mz	2.501 × 10^5^	4.520 × 10^4^
	Mp	2.51 × 10^5^	3.91 × 10^4^
Rms radius moments (nm)	Rz	1.5 nm	1.5 nm

**Table 2:** The primary chemical characteristics of MBAP-1 and MBAP-2.

Sample	Yield (%)	Total Sugar Content (%)	Uronic Acid (%)	Protein (%)	Flavonoids (%)	Monosaccharide Composition (*w*/*w*)				
						Glc	GlcA	GalA	Gal	Ara
MBAP-1	0.14	86.06 ± 2.76	9.64 ± 0.56	1.90 ± 0.15	12.03 ± 1.20	65.17 (54.54 ^#^)	6.27 (4.87 ^#^)	5.42 (4.21 ^#^)	5.03 (4.21 ^#^)	18.10 (18.18 ^#^)
MBAP-2	0.16	82.03 ± 1.77	-	2.06 ± 0.13	15.96 ± 1.36	88.18 (88.41 ^#^)	-	-	-	-

^#^ The molar ratio of each monosaccharide.

**Table 3:** The PMAAs results of MBAP-1, MBAP-1R and MBAP-2.

PMAAs	Linkages	Molar Ratio			Major Mass Fragments (*m*/*z*)
		MBAP-1	MBAP-1R	MBAP-2	
1,3,4,5,6-Penta-*O*-acetyl-1-deuterio-2-*O*-methyl-d-glucitol	1,3,4,6-linked-Glc*p*	1.00	1.00		43, 59, 87, 118, 139, 333
1,2,3,5-Tetra-*O*-acetyl-1-deuterio-4,6-di-*O*-methyl-d-glucitol	1,2,3-linked-Glc*p*	1.03	0.95		43, 59, 71, 87, 101, 129, 161, 202, 262
1,4,5-Tri-*O*-acetyl-1-deuterio-2,3-di-*O*-methyl-l-arabinitol	1,5-linked-Ara*f*	1.87	1.95		43, 59, 87, 102, 118, 129, 189
1,4-Di-*O*-acetyl-1-deuterio-2,3,5-tri-*O*-methyl-l-arabinitol	1-linked-Ara*f*	2.21	2.24		43, 59, 71, 87, 102, 118, 129, 161, 162
1,5-Di-*O*-acetyl-1-deuterio-2,3,4,6-tetra-*O*-methyl-d-galactitol	1-linked-Gal*p*	n.d.	1.12		43, 71, 87, 102, 118, 129, 145, 161, 162, 205
1,3,4,5-Tetra-*O*-acetyl-1-deuterio-2,6-di-*O*-methyl-d-glucitol	1,3,4-linked-Glc*p*	1.98	2.01	0.89	43, 59, 87, 118, 129, 160, 185, 305
1,4,5-Tri-*O*-acetyl-1-deuterio-2,3,6-tri-*O*-methyl-d-galactitol	1,4-linked-Gal*p*	1.03	0.99		43, 59, 71, 87, 102, 118, 129, 162, 233
1,4,5-Tri-*O*-acetyl-1-deuterio-2,3,6-tri-*O*-methyl-d-glucitol	1,4-linked-Glc*p*	8.01	7.89	0.35	43, 59, 71, 87, 102, 118, 129, 162, 233
1,5-Di-*O*-acetyl-1-deuterio-2,3,4,6-tetra-*O*-methyl-d-glucitol	1-linked-Glc*p*	n.d.	1.10	1.20	43, 71, 87, 102, 118, 129, 145, 161, 162, 205
1,3,5,6-Tetra-*O*-acetyl-1-deuterio-2,4-di-*O*-methyl-d-glucitol	1,3,6-linked-Glc*p*			0.38	43, 59, 87, 118, 129, 139, 160, 189, 234, 305
1,5,6-Tri-*O*-acetyl-1-deuterio-2,3,4-tri-*O*-methyl-d-glucitol	1,6-linked-Glc*p*			1.01	43, 59, 87, 99, 101, 118, 129, 162, 189, 233

Note: n.d. means not detected in GC-MS.

**Table 4:** ^1^H-NMR (600 MHz) and ^13^C-NMR (150 MHz) chemical shifts of MBAP-1 and MBAP-2.

Name	Code	Residues	Chemical Shifts (ppm)					
			H1/C1	H2/C2	H3/C3	H4/C4	H5/C5	H6/C6
MBAP-1	A	→3,4,6)-α-d-Glc*p*-(1→	5.26/101.76	3.36/72.47	4.01/86.21	3.50/77.49	3.98/71.22	3.85/69.46
	B	→2,3)-α-d-Glc*p*-(1→	5.21/94.86	4.33/84.89	4.00/80.62	3.88/72.97	3.92/73.13	3.61/61.54
	C	→5)-α-l-Ara*f*-(1→	5.13/109.84	4.11/79.08	3.99/79.21	4.11/83.30	3.78/68.53	
	D	α-l-Ara*f*-(1→	5.07/110.23	4.12/84.16	3.88/76.51	4.06/86.96	3.72/63.41	
	E	β-d-Gal*p*A-(1→	4.95/100.47	3.77/69.59	3.47/72.12	3.53/73.99	3.73/68.31	-/175.87
	F	→3,4)-β-d-Glc*p*-(1→	4.73/102.72	3.65/76.30	4.06/86.66	3.77/76.40	3.69/78.60	3.92/63.41
	G	→4)-β-d-Gal*p*-(1→	4.70/98.66	3.79/74.14	3.97/75.00	3.65/76.18	4.32/78.82	3.80/59.78
	H	→4)-β-d-Glc*p*-(1→	4.62/98.70	3.30/72.40	3.55/74.20	3.92/78.55	3.63/71.68	3.73/58.92
	I	β-d-Glc*p*A-(1→	4.48/105.32	3.50/72.33	3.60/76.54	3.62/75.58	3.59/77.17	-/178.66
MBAP-2	A	→3,6)-α-d-Glc*p*-(1→	5.32/98.91	4.40/80.62	4.31/81.47	3.76/70.88	3.84/73.76	3.71/69.56
	B	→6)-α-d-Glc*p*-(1→	5.23/98.68	4.25/79.52	3.93/73.76	3.88/70.28	3.90/74.01	3.62/68.56
	C	→3,4)-α-d-2-OCH_3_-Glc*p*-(1→	5.08/100.60	3.67/75.51	4.39/82.41	3.76/74.51	3.78/74.77	3.53/60.32
	D	→4)-β-d-Glc*p*-(1→	4.92/100.26	3.62/70.51	3.48/72.21	3.64/74.82	3.35/72.40	3.74/60.23
	E	β-d-Glc*p*-(1→	4.37/102.53	3.75/73.10	3.56/73.52	3.40/70.22	3.46/75.83	3.68/60.48

Note: The ^1^H and ^13^C chemical shifts of -OMe group were 3.71 and 55.20 ppm.

## Data Availability

Data will be provided upon request.

## Supplementary Materials

The following supporting information can be downloaded at: https://www.mdpi.com/article/10.3390/molecules27144532/s1. Methods S1: Materials; Methods S2: Homogeneity and molecular weight determination; Methods S3: Monosaccharide analysis of MBAP-2; Methods S4: Reduction, methylation and GC-MS analysis; Methods S5: NMR spectroscopy analysis; Table S1: The detailed setting parameters in NMR experiments; Figure S1: The HPGPC-ELSD results of MBAP-1 and MBAP-2; Figure S2: The GC-MS spectrogram of monosaccharide of MBAP-2; Figure S3: FT-IR spectroscopy analysis of MBAP-1 and MBAP-2; Figure S4: The mass spectra of PMAAs of MBAP-1; Figure S5: The mass spectra of PMAAs of MBAP-2; Figure S6: The ^1^H- and ^13^C-NMR spectra of MBAP-1; Figure S7: The ^1^H- and ^13^C-NMR spectra of MBAP-2; Figure S8: The ^1^H-NMR and ^13^C-NMR spectra of quercetin in d_6_-DMSO and d_6_-DMSO: D_2_O (1:1).; Figure S9: The AFM results of MBAP-1 and MBAP-2. References [45,46] are cited in the Supplementary Materials.

## Sample Availability

Samples of the MBAP-1 and MBAP-2 are available from the authors.
