# Supplementary material for: Two Natural Flavonoid Substituted Polysaccharides from Tamarix chinensis: Structural Characterization and Anticomplement Activities
Source: Molecules. 2022 Jul 15;27(14):4532. doi: 10.3390/molecules27144532 (PMC9315555; doi:10.3390/molecules27144532)
Supplement: Supplementary file 1 [file molecules-27-04532-s001.zip › molecules-1823444-supplementary.pdf]

molecules

Supplementary Materials

Two Natural Flavonoid Substituted Polysaccharides from *Tamarix chinensis*: Structural Characterization and Anticomplement Activities

Yukun Jiao, Yiting Yang, Lishuang Zhou, Daofeng Chen * and Yan Lu *

School of Pharmacy, Institutes of Integrative Medicine, Fudan University, Shanghai, 201203, China; 17111030034@fudan.edu.cn (Y.J.); 17211030024@fudan.edu.cn (Y.Y.); 20111030064@fudan.edu.cn (L.Z.)
* Correspondence: dfchen@shmu.edu.cn (D.C.); luyan@fudan.edu.cn (Y.L.)

Supplementary methods

S1. Materials

Monosaccharide standards, $NaBH_4$, D_2O, trifluoroacetic acid (TFA), 1-pheny-3-methyl-5-pyrazolone (PMP), *N*-cyclohexyl-*N*-(2-morpholinoehtyl) carbodiimidemetho-*p*-toluenesul-phonate (CMC) and anhydrous DMSO were purchased from Sigma-Aldrich (St. Louis, MO, USA). L-cysteine methyl ester, *O*-tolyl isothiocyanate heparin and quercetin CRS (purity ≥ 98%) were purchased from Macklin Bio-chem Co. Ltd (Shanghai, China). Packing material DEAE-cellulose and Sephacryl S-200 were purchased from GE Healthcare Bio-Sciences (Piscataway, NJ, USA).

The barbital buffered saline was purchased from Beijing leagene biotech. Co. Ltd (Beijing, China). Sheep erythrocytes (EAs) were purchased from Shanghai Shengwei biotech. Co. Ltd (Shanghai, China). ABTS and FARP Elisa kits were purchased from Beyotime biotech. Co. Ltd (Shanghai, China). Guinea pigs and New Zealand white rabbits were purchased from the Laboratory Animals Research Institute of Fudan University, Shanghai, China. Guinea pig sera (GPS) were obtained from Guinea pigs and anti-sheep erythrocyte antibody was from rabbit antiserum which was provided by Associate Prof. Yunyi Zhang (School of Pharmacy, Fudan University).

S2. Homogeneity and Molecular Weight Determination

The homogeneity of MBAP-1 and MBAP-2 was analyzed on an Agilent 1260 HPLC system using the G5000 PW_{XL} (7.6 mm × 300 mm, i.d. 10 μm, TOSOH, Tokyo, Japan) and G3000 PW_{XL} columns (7.6 mm × 300 mm, i.d. 7 μm, TOSOH, Tokyo, Japan) in series and eluted with water at 0.5 mL/min, detected with Agilent 1260 infinity ELSD. The drift tube temperature of ELSD was set at 100 °C, and the nitrogen pressure was 3.0 bar. The molecular weight (Mw) was determined by high performance size-exclusion chromatography combined with multi-angle laser photometry, as well as using a refractive index system (HPSEC-MALLS-RI) (DAWN HELEOS II, Wyatt, Santa Barbara, CA, USA) according to the previous literature [1]. The data were analyzed using Wyatt ASTRA 6.1.5.22 software (Wyatt, Santa Barbara, CA, USA).

S3. Monosaccharide Analysis of MBAP-2

The unknown monosaccharide in MBAP-2 was analyzed by GC-MS [2]. Briefly, MBAP-2 was firstly fully hydrolyzed with TFA (4 M) at 110 °C for 2 h, then reduced by $NaBD_4$ and acetylated by acetic anhydride/pyridine. Finally, the alditol acetates were analyzed by GC-MS (QP 2010 Ultra, Shimadu, Japan) attached to a HP-5MS column (0.25 mm × 30 m, i.d. 0.25 μm, Thermo, Waltham, MA, USA). The partially methylated alditol acetates (PMAAs) were assigned by ion fragment analysis.

S4. Reduction, Methylation and GC-MS Analysis

First, MBAP-1 were reduced three times with CMC-NaBH4 using the method described before [3]. Furthermore, the reduced product of MBAP-1 (labeled as MBAP-1R), MBAP-1, and MBAP-2 were methylated according to the Hakomori method with some modifications. In brief, MBAP-1R, MBAP-1 and MBAP-2 were dissolved in DMSO, methylated with CH_3I, hydrolyzed using TFA, reduced by NaBD4 and acetylated by acetic anhydride/pyridine. The derivative products were analyzed by GC-MS (QP 2010 Ultra, Shimadzu, Kyoto, Japan) attached to a TG-5MS column (0.25 mm × 30 m, i.d. 0.25 μm, Thermo, Waltham, MA, USA). The operation procedures of GC analysis were set as follows: injection and detector temperatures were both 250 °C. The column temperature procedure was set as follows: initial 150 °C and holding for 5 min, increasing at 2 °C/min to 220 °C, holding for 10 min, and then raised at 5 °C/min to 240 °C, holding for 5 min. The partially methylated alditol acetates (PMAAs) were assigned by comparison with retention time, previous reports, and the complex carbohydrate research center spectral database (CCRC, https://glygen.ccrc.uga.edu/ccrc/specdb/ms/pmaa/pframe.html#na), and their molar ratios were evaluated by the peak areas.

S5. NMR Spectroscopy Analysis

The ^{1}H-NMR, ^{13}C-NMR, HSQC and HMBC spectra were recorded at 24 °C with an NMR spectrometer (600 M, AVANCE III HD, Bruker, Fällanden, Switzerland). The detailed parameters are summarized in Table S1.

Supplementary Table and Figure captions

Table S1. The detailed setting parameters of MBAP-1 and MBAP-2 in NMR experiments.

Figure S1. The HPGPC-ELSD results of MBAP-1 (**A**) and MBAP-2 (**B**).

Figure S2. The GC-MS spectrogram of monosaccharide of MBAP-2.

Figure S3. FT-IR spectroscopy analysis of MBAP-1 and MBAP-2. (**A**) The spectrum of MBAP-1. (**B**) The spectrum of MBAP-2.

Figure S4. The mass spectra of PMAAs of MBAP-1.

Figure S5. The mass spectra of PMAAs of MBAP-2.

Figure S6. The ^{1}H-NMR (**A**) and ^{13}C-NMR (**B**) spectra of MBAP-1.

Figure S7. The ^{1}H-NMR (**A**) and ^{13}C-NMR (**B**) spectra of MBAP-2.

Figure S8. The ^{1}H-NMR and ^{13}C-NMR spectra of quercetin in d_6-DMSO and d_6-DMSO: D_2O (1:1).

Figure S9. The AFM results of MBAP-1 and MBAP-2.

Supplementary Table

Table S1. The detailed setting parameters of MBAP-1 and MBAP-2 in NMR experiments.

Sample Name	Experiment	Spectrometer Frequency (MHz)	Spectral Width	Number of Scans	Acquisition Time
MBAP-1	^{1}H	600.13	12019.2	64	1.3631
	^{13}C	150.9	33333.3	14943	0.983
	HSQC	(600.13, 150.90)	(6313.1, 26455.0)	128	0.0811
	HMBC	(600.13, 150.90)	(6313.1, 33112.6)	928	0.1622
MBAP-2	^{1}H	600.13	12019.2	64	1.3631
	^{13}C	150.9	36231.9	10240	0.9044
	HSQC	(600.13, 150.90)	(7812.5, 24875.6)	128	0.1311
	HMBC	(600.13, 150.90)	(6313.1, 33112.6)	192	0.1622

Supplementary Figures

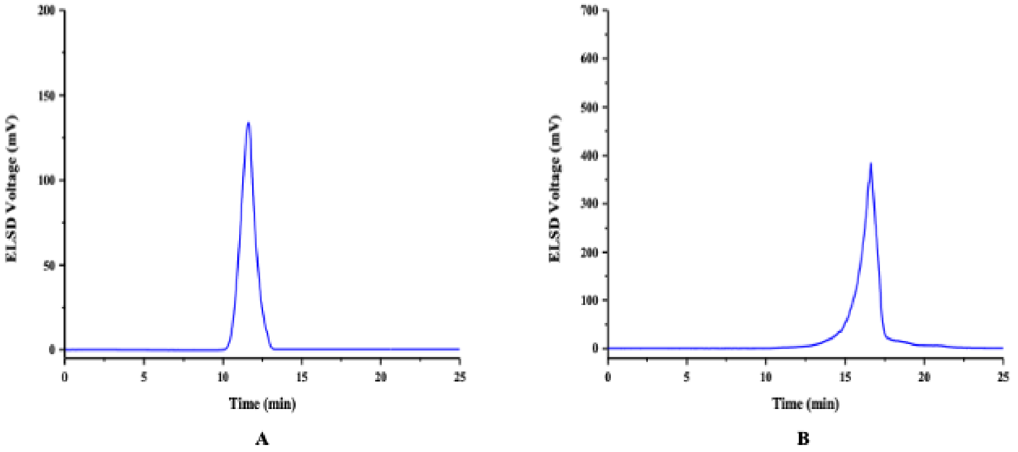

Figure S1. The HPGPC-ELSD results of MBAP-1 (**A**) and MBAP-2 (**B**).

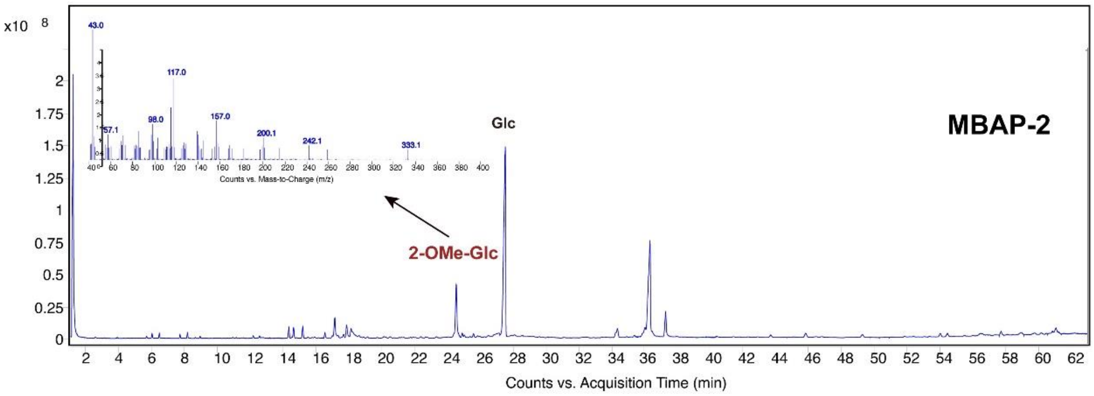

Figure S2. The GC-MS spectrogram of monosaccharide of MBAP-2.

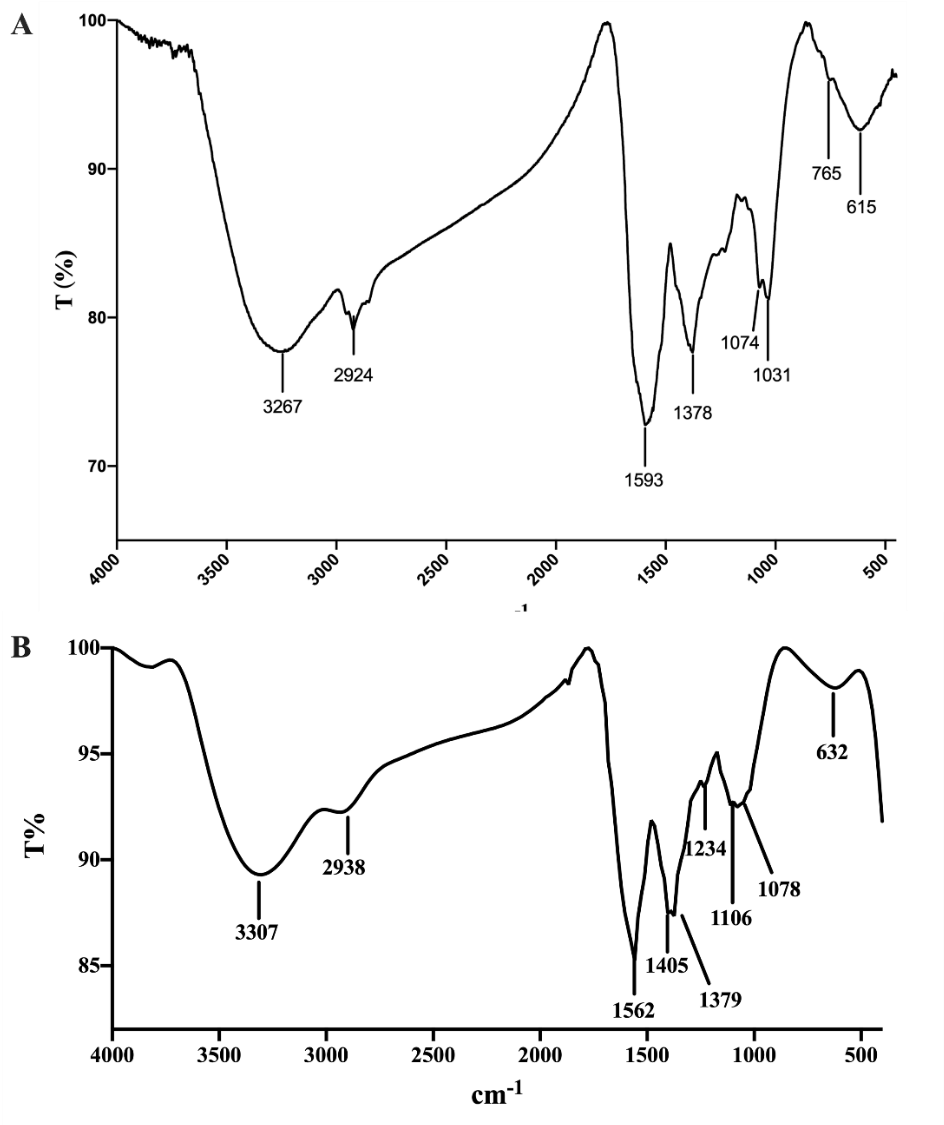

Figure S3. FT-IR spectroscopy analysis of MBAP-1 and MBAP-2. (**A**) The spectrum of MBAP-1. (**B**) The spectrum of MBAP-2.

A

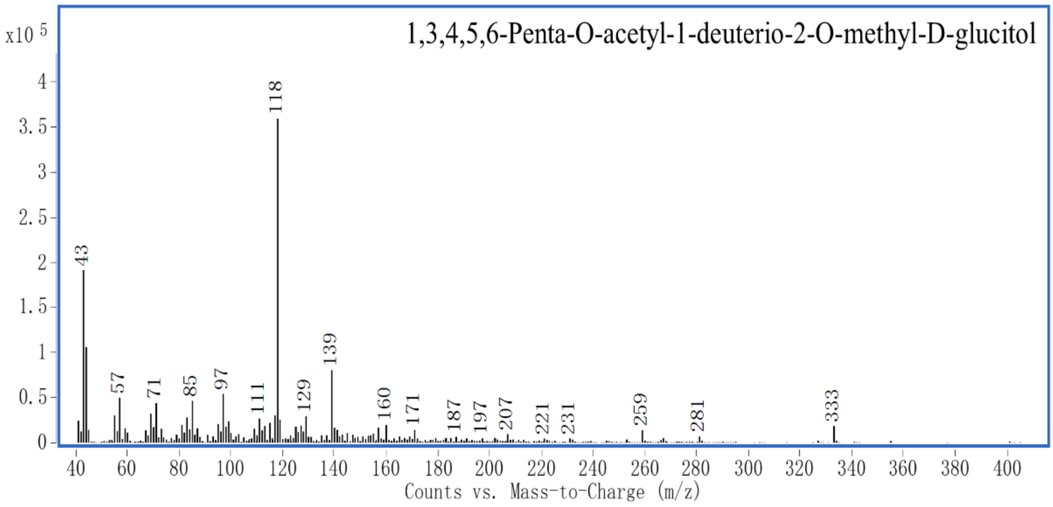
1,3,4,5,6-Penta-O-acetyl-1-deuterio-2-O-methyl-D-glucitol
x10 5
43
57
71
85
97
111
118
129
139
160
171
187
197
207
221
231
259
281
333
Counts vs. Mass-to-Charge (m/z)

B

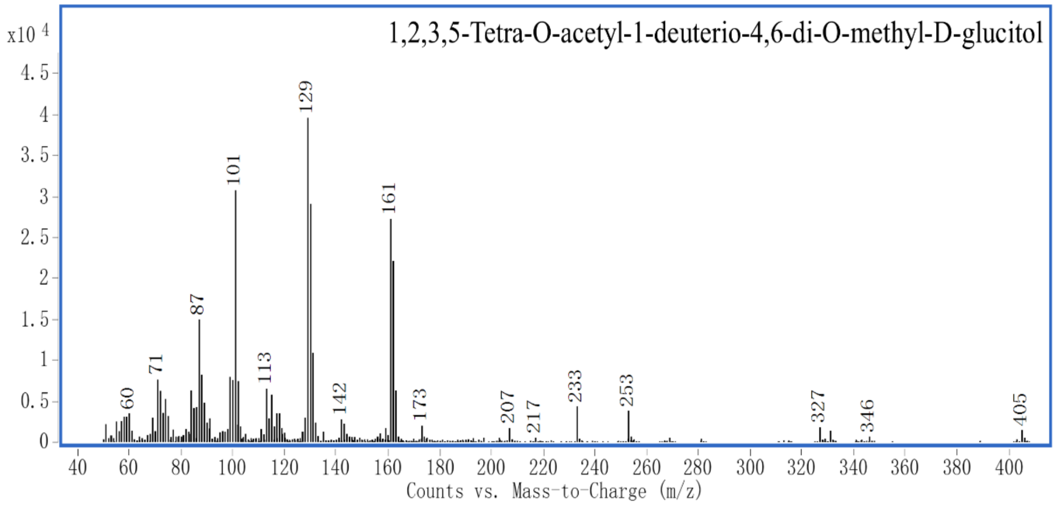
1,2,3,5-Tetra-O-acetyl-1-deuterio-4,6-di-O-methyl-D-glucitol
x10 4
60
71
87
101
113
129
142
161
173
207
217
233
253
327
346
405
Counts vs. Mass-to-Charge (m/z)

C

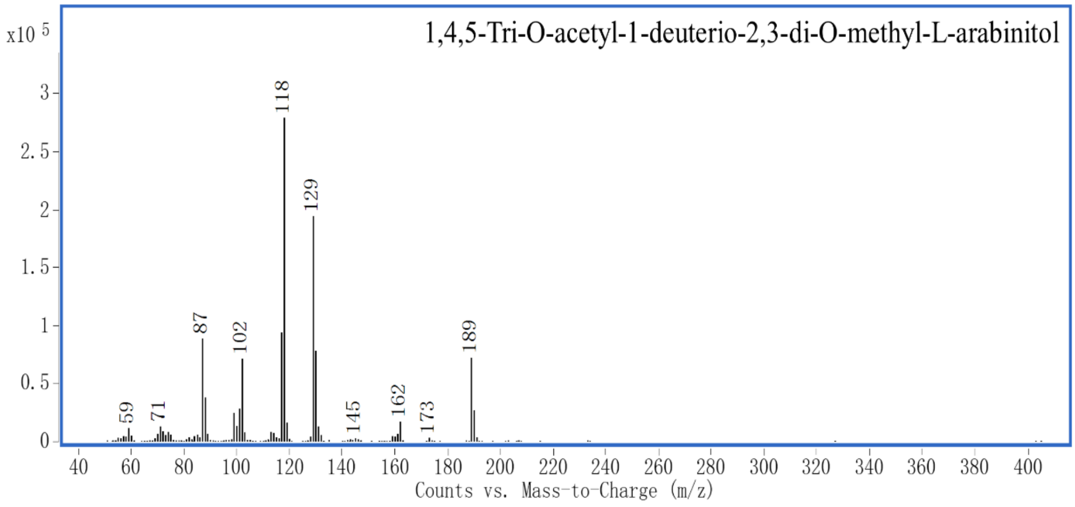
1,4,5-Tri-O-acetyl-1-deuterio-2,3-di-O-methyl-L-arabinitol
x10 5
59
71
87
102
118
129
145
162
173
189
Counts vs. Mass-to-Charge (m/z)

D

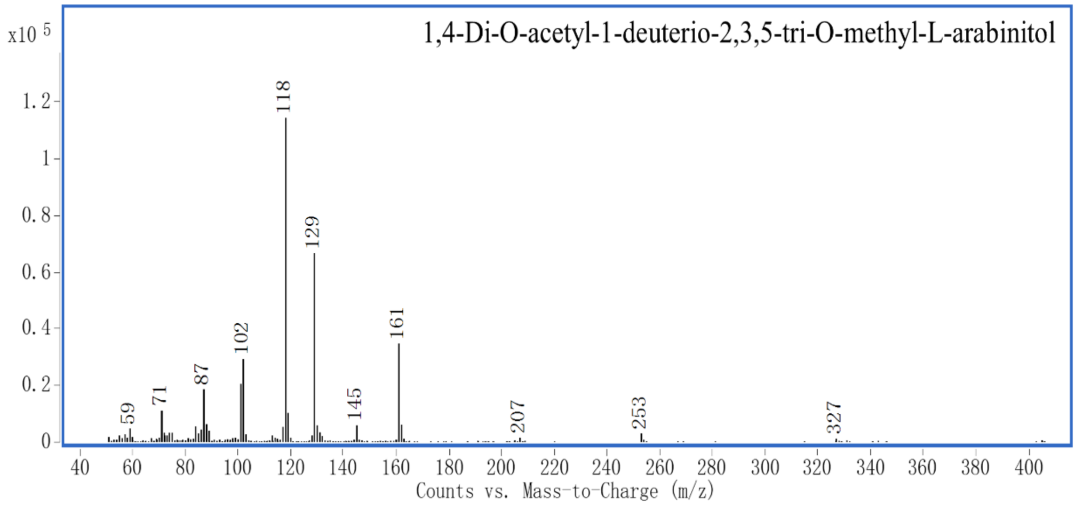
1,4-Di-O-acetyl-1-deuterio-2,3,5-tri-O-methyl-L-arabinitol
x10 5
1.2
1
0.8
0.6
0.4
0.2
0
59
71
87
102
118
129
145
161
207
253
327
40 60 80 100 120 140 160 180 200 220 240 260 280 300 320 340 360 380 400
Counts vs. Mass-to-Charge (m/z)

E

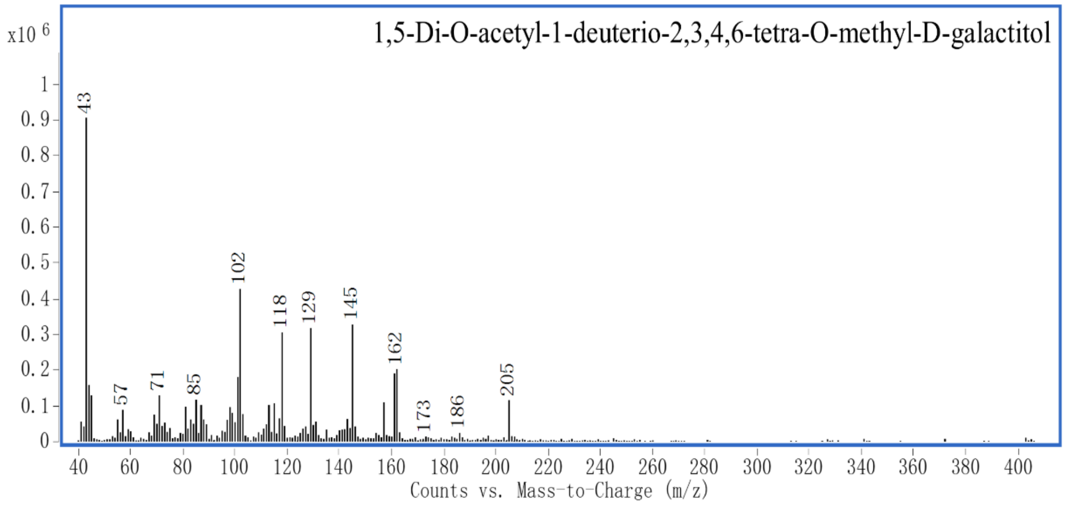
1,5-Di-O-acetyl-1-deuterio-2,3,4,6-tetra-O-methyl-D-galactitol
x10 6
1
0.9
0.8
0.7
0.6
0.5
0.4
0.3
0.2
0.1
0
43
57
71
85
102
118
129
145
162
173
186
205
40 60 80 100 120 140 160 180 200 220 240 260 280 300 320 340 360 380 400
Counts vs. Mass-to-Charge (m/z)

F

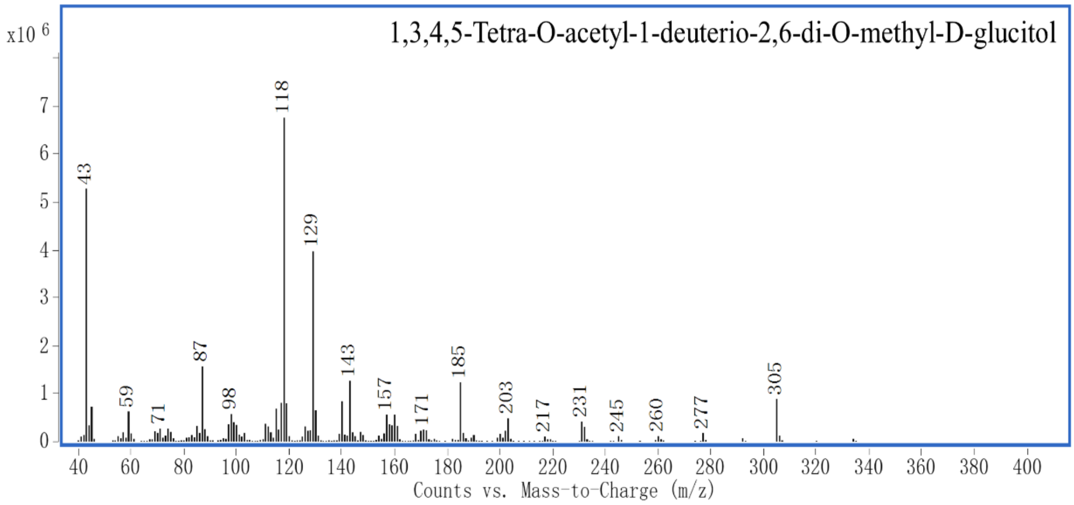
1,3,4,5-Tetra-O-acetyl-1-deuterio-2,6-di-O-methyl-D-glucitol
x10 6
7
6
5
4
3
2
1
0
43
59
71
87
98
118
129
143
157
171
185
203
217
231
245
260
277
305
40 60 80 100 120 140 160 180 200 220 240 260 280 300 320 340 360 380 400
Counts vs. Mass-to-Charge (m/z)

G

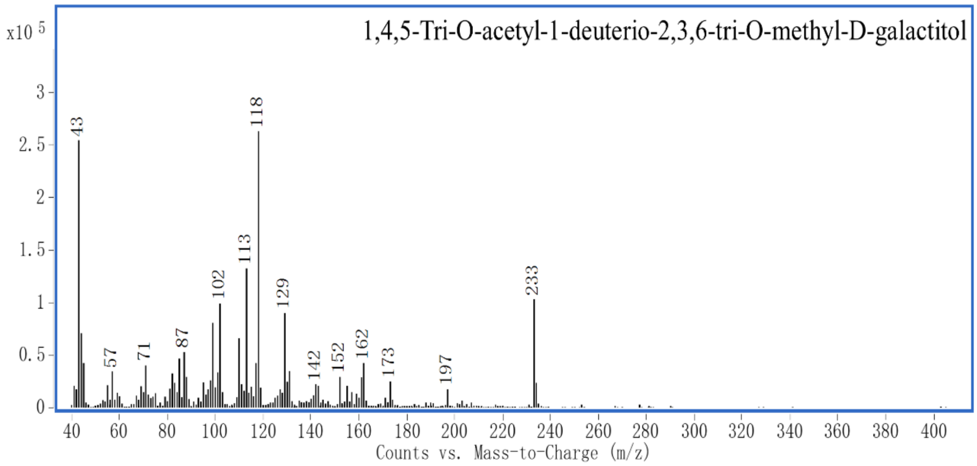

H

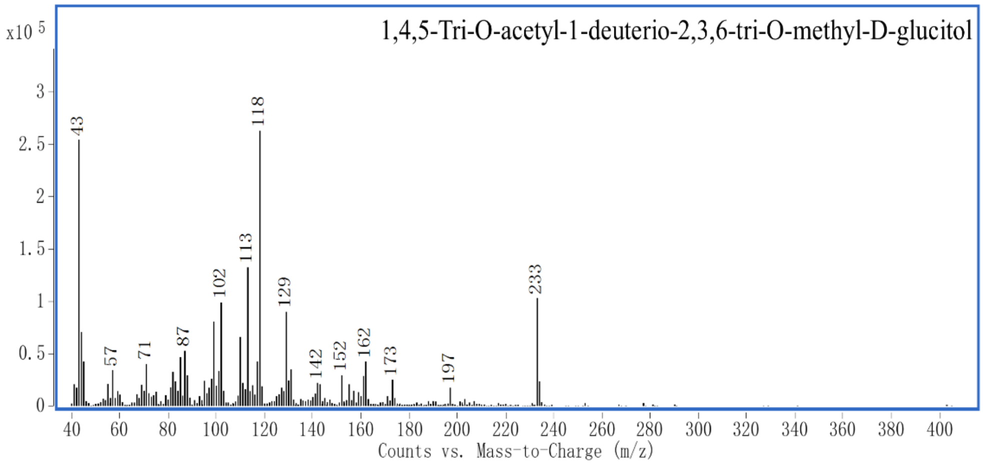

I

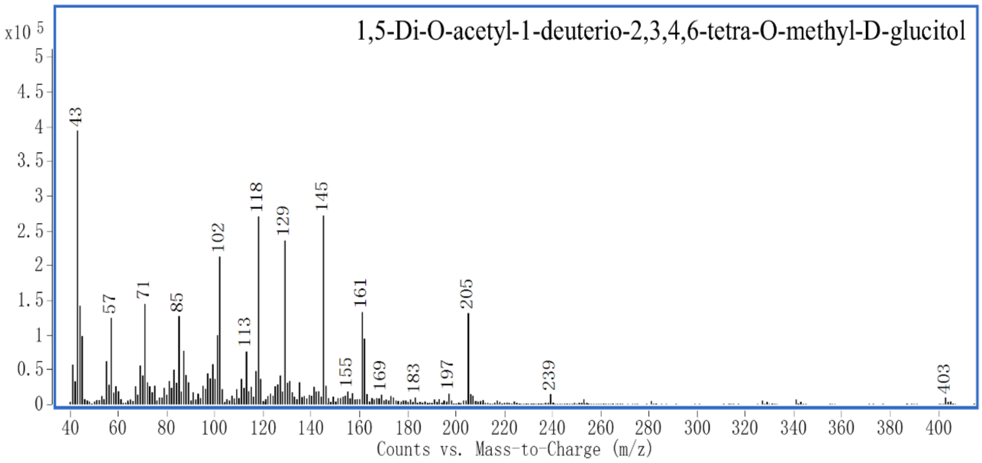

Figure S4. The mass spectra of PMAAs of MBAP-1. (**A**) 1,3,4,5,6-Penta-*O*-acetyl-1-deuterio-2-*O*-methyl-D-glucitol, (**B**) 1,2,3,5-Tetra-*O*-acetyl-1-deuterio-4,6-di-*O*-methyl-D-glucitol, (**C**) 1,4,5-Tri-*O*-acetyl-1-deuterio-2,3-di-*O*-methyl-D-arabinitol, (**D**) 1,4-Di-*O*-acetyl-1-deuterio-2,3,5-tri-*O*-methyl-L-arabinitol, (**E**) 1,5-Di-*O*-acetyl-1-deuterio-2,3,4,6-tetra-*O*-methyl-D-galactitol, (**F**) 1,3,4,5-Tetra-*O*-acetyl-1-deuterio-2,6-di-*O*-methyl-D-glucitol, (**G**) 1,4,5-Tri-*O*-acetyl-1-deuterio-2,3,6-tri-*O*-methyl-D-galactitol, (**H**) 1,4,5-Tri-*O*-acetyl-1-deuterio-2,3,6-tri-*O*-methyl-D-glucitol, (**I**) 1,5-Di-*O*-acetyl-1-deuterio-2,3,4,6-tetra-*O*-methyl-D-glucitol.

A

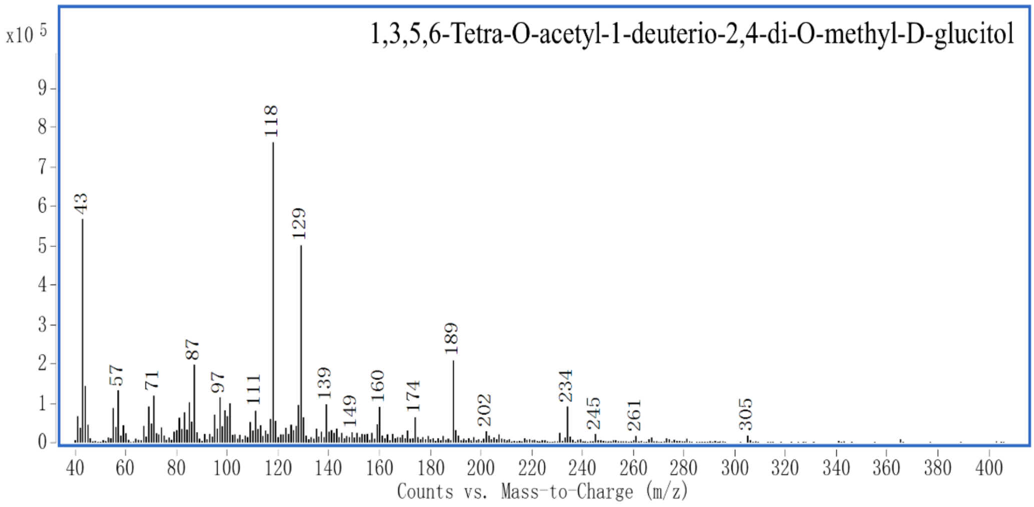

1,3,5,6-Tetra-O-acetyl-1-deuterio-2,4-di-O-methyl-D-glucitol
x10 5
Counts vs. Mass-to-Charge (m/z)
43
57
71
87
97
111
118
129
139
149
160
174
189
202
234
245
261
305

B

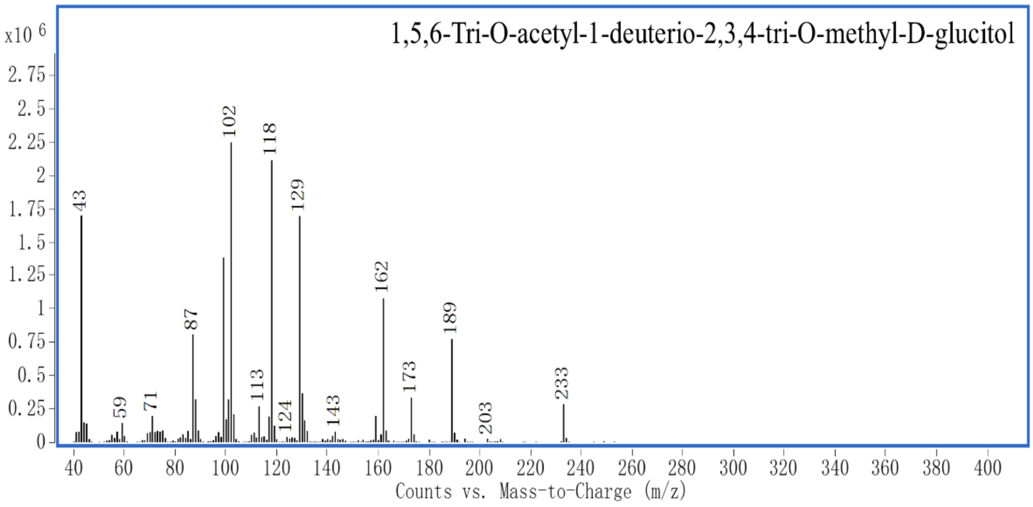

1,5,6-Tri-O-acetyl-1-deuterio-2,3,4-tri-O-methyl-D-glucitol
x10 6
Counts vs. Mass-to-Charge (m/z)
43
59
71
87
102
113
118
124
129
143
162
173
189
203
233

C

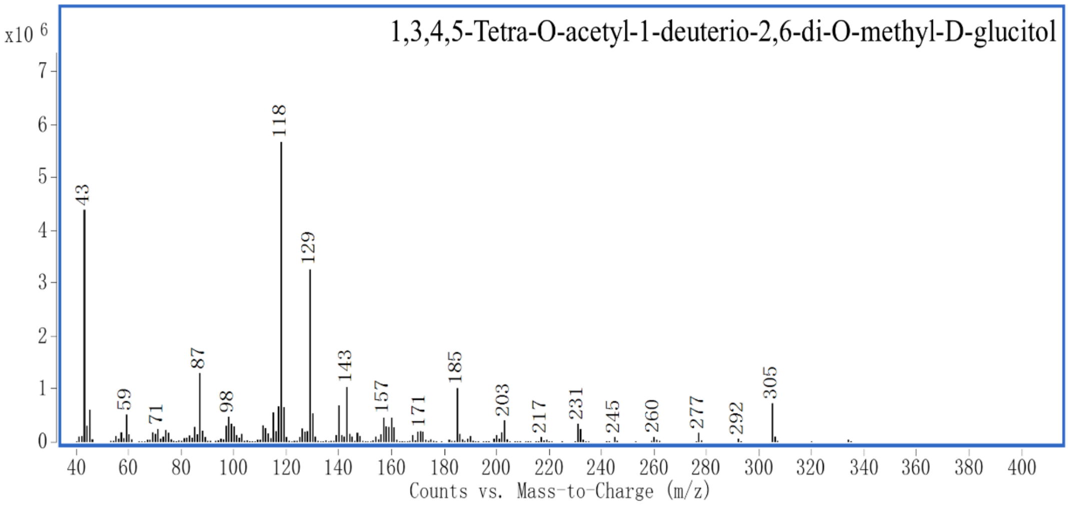

1,3,4,5-Tetra-O-acetyl-1-deuterio-2,6-di-O-methyl-D-glucitol
x10 6
Counts vs. Mass-to-Charge (m/z)
43
59
71
87
98
118
129
143
157
171
185
203
217
231
245
260
277
292
305

D

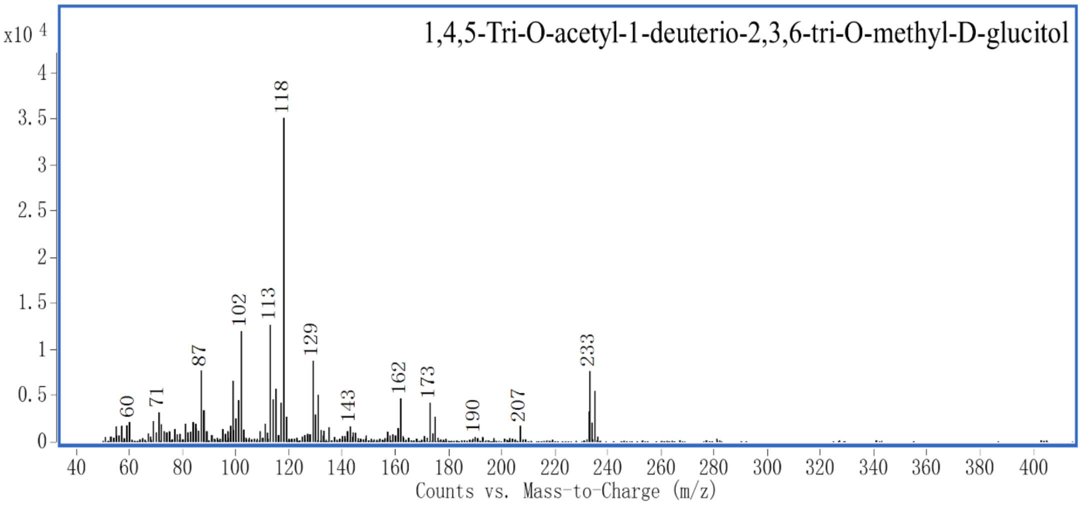

E

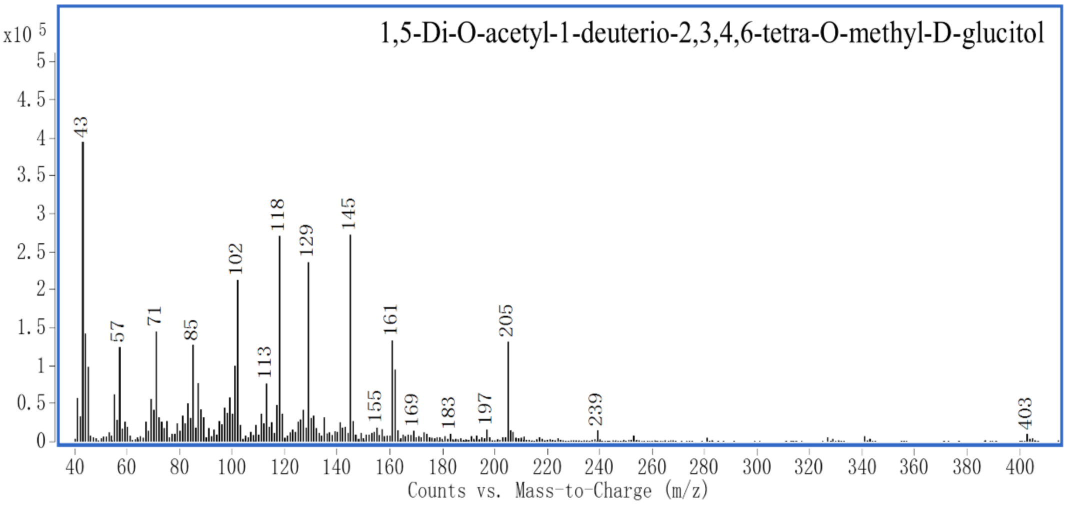

Figure S5. The mass spectra of PMAAs of MBAP-2. (**A**) 1,3,5,6-Tetra-*O*-acetyl-1-deuterio-2,4-di-*O*-methyl-D-glucitol, (**B**) 1,5,6-Tri-*O*-acetyl-1-deuterio-2,3,4-tri-*O*-methyl-D-glucitol, (**C**) 1,3,4,5-Tetra-*O*-acetyl-1-deuterio-2,6-di-*O*-methyl-D-glucitol, (**D**) 1,4,5-Tri-*O*-acetyl-1-deuterio-2,3,6-tri-*O*-methyl-D-glucitol, (**E**) 1,5-Di-*O*-acetyl-1-deuterio-2,3,4,6-tetra-*O*-methyl-D-glucitol.

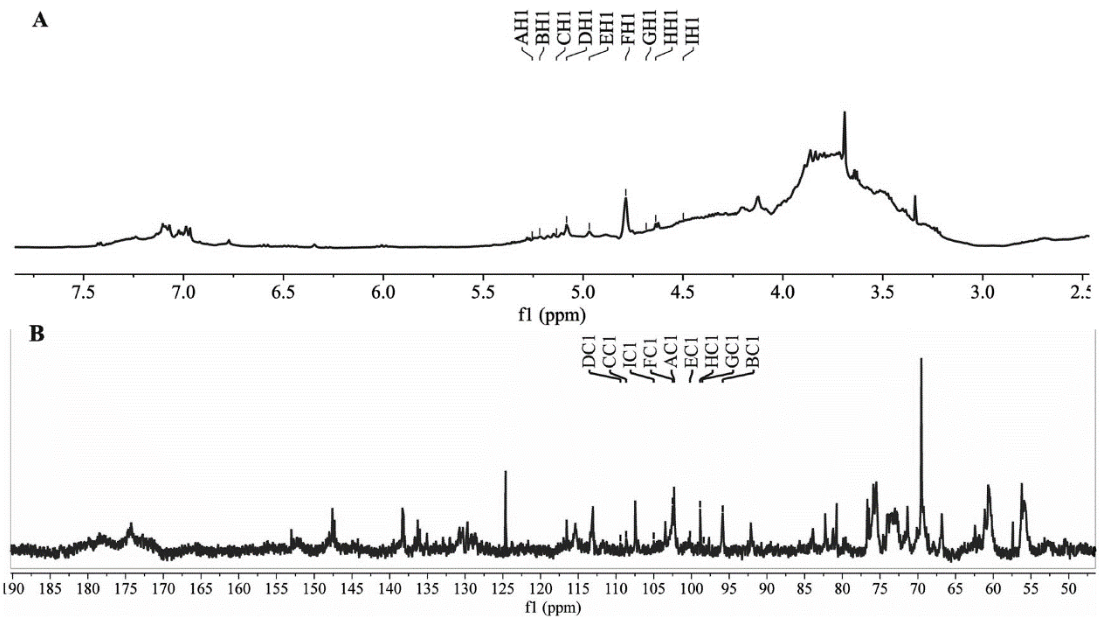

Figure S6. The ^{1}H-NMR (**A**) and ^{13}C-NMR (**B**) spectra of MBAP-1.

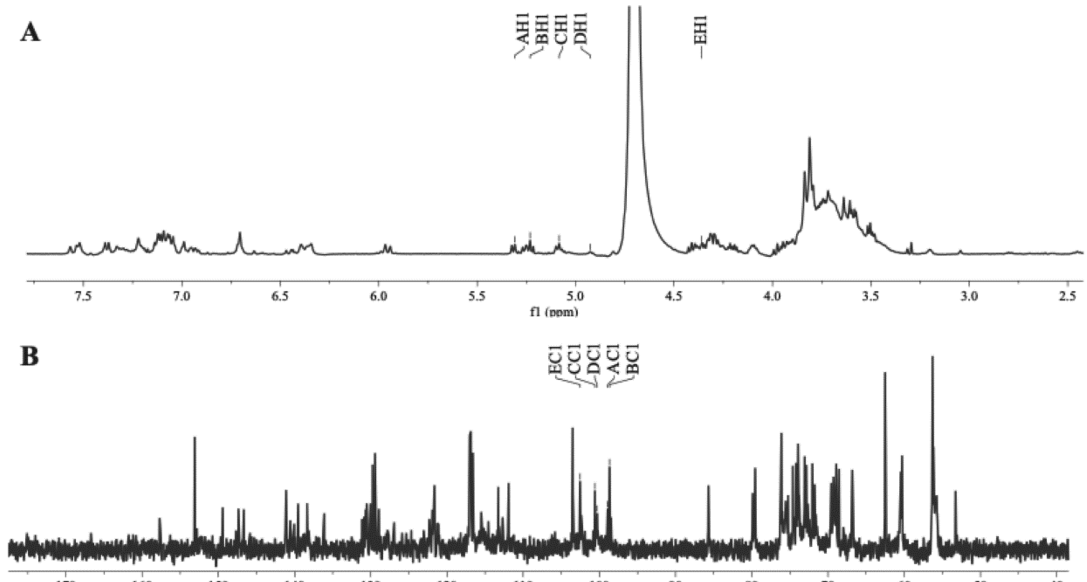

Figure S7. The ^{1}H-NMR (**A**) and ^{13}C-NMR (**B**) spectra of MBAP-2.

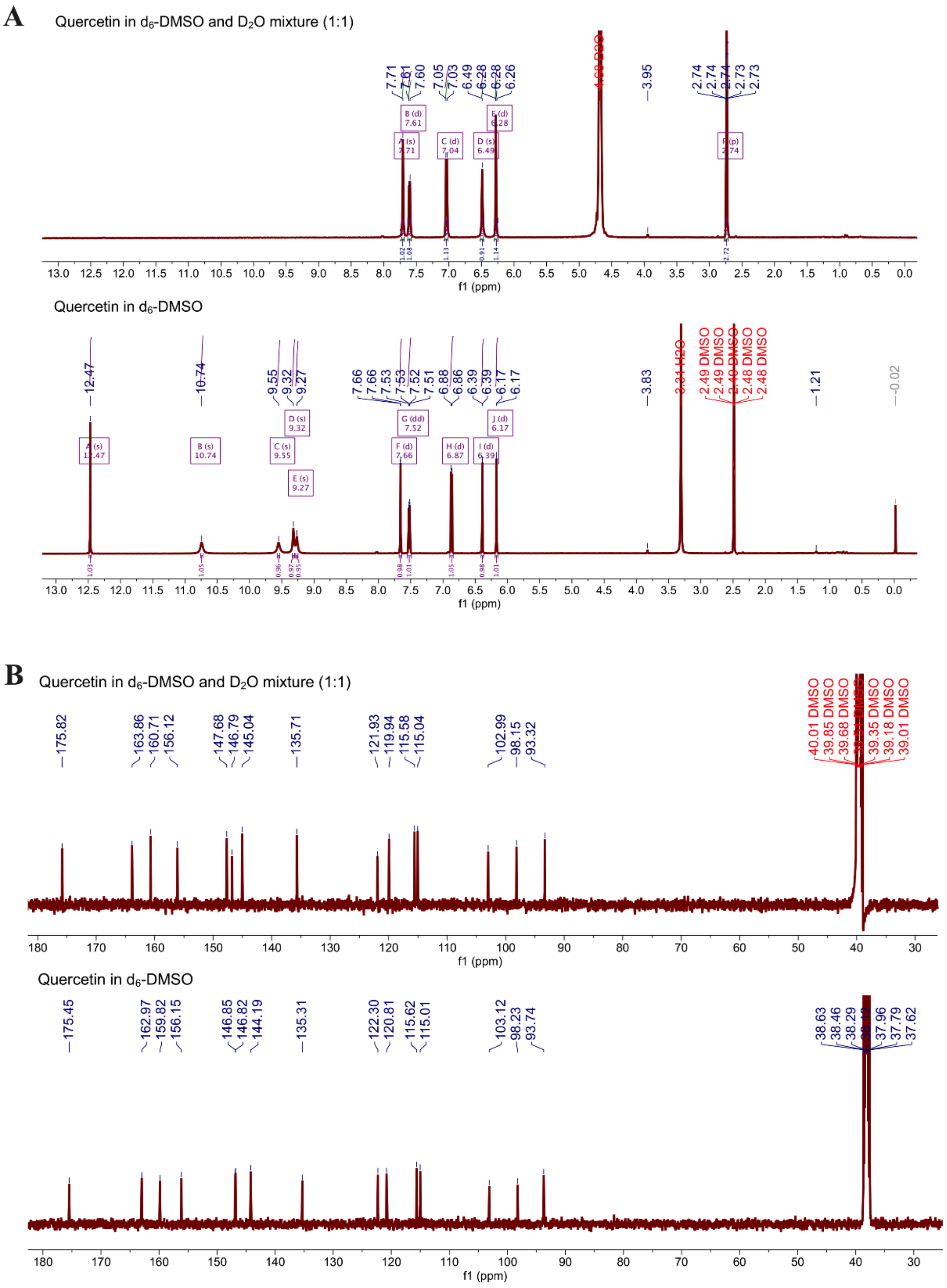

Figure S8. The ^{1}H-NMR and ^{13}C-NMR spectra of quercetin in d_6-DMSO and d_6-DMSO: D_2O (1:1).

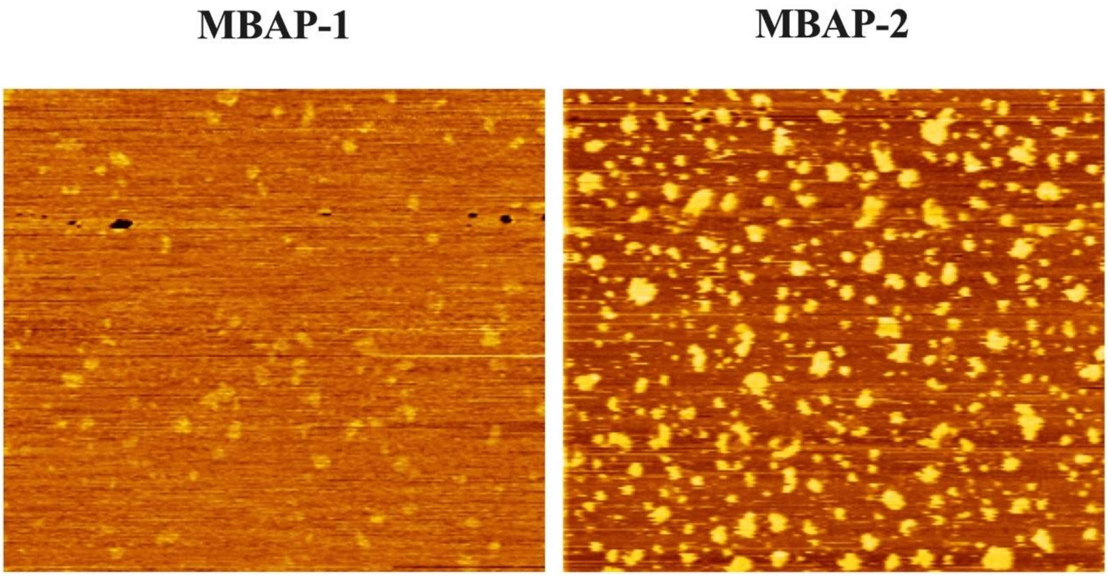

Figure S9. The AFM results of MBAP-1 and MBAP-2.

References

1. Zhang, S.J.; Zhang, Q.; An, L.J.; Zhang, J.J.; Li, Z.G.; Zhang, J.; Li, Y.H.; Tuerhong, M.; Ohizumi, Y.; Jin, J.; et al. A fructan from *Anemarrhena asphodeloides* Bunge showing neuroprotective and immunoregulatory effects. *Carbohyd. Polym.* **2020**, *229*, 115477.
2. Chen, X.; Cao, D.; Zhou, L.; Jin, H.; Dong, J.; Yao, J.; Ding, K. Structure of a polysaccharide from *Gastrodia elata* Bl., and oligosaccharides prepared thereof with anti-pancreatic cancer cell growth activities. *Carbohyd. Polym*. **2020**, *86*, 1300–1305.
3. Huo, J.; Lu, Y.; Jiao, Y.; Chen, D. Structural characterization and anticomplement activity of an acidic polysaccharide from *Hedyotis diffusa*. *Int. J. Biol. Macromol.* **2020**, *155*, 1553–1560.
